# Emergence and rapid dissemination of highly resistant NDM-14-producing *Klebsiella pneumoniae* ST147, France, 2022

**DOI:** 10.2807/1560-7917.ES.2023.28.42.2300095

**Published:** 2023-10-19

**Authors:** Cécile Emeraud, Aba Mahamat, Agnès B. Jousset, Sandrine Bernabeu, Tania Goncalves, Camille Pommier, Delphine Girlich, Aurélien Birer, Christophe Rodriguez, Jean-Michel Pawlotsky, Thierry Naas, Rémy A. Bonnin, Laurent Dortet

**Affiliations:** 1 Department of Bacteriology-Hygiene, Bicêtre Hospital, Assistance Publique - Hôpitaux de Paris, Le Kremlin-Bicêtre, France; 2 UMR-1184, INSERM, University Paris-Saclay, CEA, Faculty of Medicine, Le Kremlin-Bicêtre, France; 3 French National Reference Center for Antibiotic Resistance, Le Kremlin-Bicêtre, France; 4 Corsica Centre for Healthcare-Associated Infections Control and Prevention, Hôpital Eugénie, Ajaccio, France; 5 Centre National de Référence de la Résistance aux antibiotiques, Service de Bactériologie, CHU Gabriel-Montpied, Clermont-Ferrand, France; 6 Université Paris-Est-Créteil (UPEC), Créteil, France; 7 Department of Virology, Hôpitaux Universitaires Henri Mondor, Assistance Publique-Hôpitaux de Paris, Créteil, France; 8 INSERM U955, Team Viruses, Hepatology, Cancer, Créteil, France

**Keywords:** New Delhi metallo-β-lactamase, high-risk clone, NDM-14, emergence, France

## Abstract

**Background:**

Since 2021, an emergence of New Delhi metallo-β-lactamase (NDM)-14-producing *Klebsiella pneumoniae* has been identified in France. This variant with increased carbapenemase activity was not previously detected in Enterobacterales.

**Aim:**

We investigated the rapid dissemination of NDM-14 producers among patients in hospitals in France.

**Methods:**

All NDM-14-producing non-duplicate clinical isolates identified in France until June 2022 (n = 37) were analysed by whole genome sequencing. The phylogeny of NDM-14-producers among all *K. pneumoniae* sequence type (ST) 147 reported in France since 2014 (n = 431) was performed. Antimicrobial susceptibility testing, conjugation experiments, clonal relationship and molecular clock analysis were performed.

**Results:**

The 37 NDM-14 producers recovered in France until 2022 belonged to *K. pneumoniae* ST147. The dissemination of NDM-14-producing *K. pneumoniae* was linked to a single clone, likely imported from Morocco and responsible for several outbreaks in France. The gene *bla*_NDM-14_ was harboured on a 54 kilobase non-conjugative IncFIB plasmid that shared high homology with a known *bla*_NDM-1_-carrying plasmid. Using Bayesian analysis, we estimated that the NDM-14-producing *K. pneumoniae* ST147 clone appeared in 2020. The evolutionary rate of this clone was estimated to 5.61 single nucleotide polymorphisms per genome per year. The NDM-14 producers were highly resistant to all antimicrobials tested except to colistin, cefiderocol (minimum inhibitory concentration 2 mg/L) and the combination of aztreonam/avibactam.

**Conclusion:**

Highly resistant NDM-14 producing *K. pneumoniae* can rapidly spread in healthcare settings. Surveillance and thorough investigations of hospital outbreaks are critical to evaluate and limit the dissemination of this clone.

**Key public health message**

**What did you want to address in this study?**

Carbapenems are broad-spectrum antimicrobials, effective against many types of bacteria, including bacteria resistant to many other antimicrobials. We wanted to investigate the emergence and rapid dissemination of a bacterium highly resistant to carbapenems (NDM-14-producing *Klebsiella pneumoniae*) in France in 2022.

**What have we learnt from this study?**

All NDM-14-producing *K. pneumoniae* isolates were closely related and part of the same cluster.

These strains were likely imported to France from Morocco, particularly the Casablanca region, where the variant emerged in 2020. These bacteria caused several outbreaks in France suggesting high-risk properties of dissemination in hospitals. Aztreonam associated with avibactam or clavulanate remain an accurate therapeutic option for infections of this highly resistant bacterium.

**What are the implications of your findings for public health?**

To limit the spread of this highly resistant clone in Europe, particular attention must be taken for patients that are directly repatriated from regions where NDM-producing bacteria occur (e.g. Morocco in our study). Our results highlight the remaining crucial role of epidemiological investigation as complement of whole genome comparison to decipher outbreaks.

## Introduction

Carbapenems remain one of the last-line options to treat infections caused by highly resistant Enterobacterales, but production of carbapenemase enzymes is one of the main resistance mechanisms to carbapenems in these bacteria. Accordingly, the increasing dissemination of carbapenemase-producing Enterobacterales (CPEs) poses a global threat to public health. Several carbapenemase types have been identified in three of the four classes of the Ambler classification: class A carbapenemases (mostly *Klebsiella pneumoniae* carbapenemases (KPC)) [1], class B carbapenemases or metallo-β-lactamases (MBLs) (mostly New Delhi MBL (NDM), Verona integron‒encoded MBL (VIM) and imipenemase (IMP)) [2] and class D carbapenemases (mostly oxacillinase (OXA)-48-types) [3]. In France, the most common carbapenemases are OXA-48-like enzymes [4], but a significant increase of NDM-producers has been observed worldwide during the last few years [5,6].

Among the MBLs, NDM, an enzyme composed of 270 amino acids, is the most common enzyme isolated worldwide. The first report of NDM-1 was from an isolate of *K. pneumoniae* recovered from a Swedish patient repatriated from New Delhi [7]. In 2022, according to the β-lactamase Database (BLDB) (http://www.bldb.eu) [8], 41 variants of NDM have been identified. The NDM-14 variant differs from NDM-1 by one amino acid substitution at position 130 (D130G). This substitution is known to increase carbapenemase activity compared with NDM-1 [9]. This variant was identified in a clinical isolate of *Acinetobacter lwoffii* from a wound of a patient hospitalised in intensive care unit in Jinan, China [9].

Although NDM has been identified in many species of Enterobacterales, *Pseudomonas* or *Acinetobacter*, it is more common in *K. pneumoniae* and *Escherichia coli* [5,6]. This carbapenemase, NDM, has been described in several *K. pneumoniae* sequence types (ST)s. However, four STs (ST11, ST14, ST15 and ST147) have been identified in outbreaks caused by NDM-producing *K. pneumoniae* [6,10,11]. Furthermore, genes conferring carbapenem resistance (*bla*_NDM_ genes) are largely plasmid-borne and the horizontal transfer is mediated by multiple plasmid types, which are most often conjugative [6].

The major burden of antimicrobial resistance is often due to the spread of high-risk clones harbouring multiple resistance genes. Among *K. pneumoniae*, isolates belonging to ST147 have been frequently identified forming high-risk multidrug-resistant clones. Indeed, *K. pneumoniae* ST147 strains have been described worldwide except Antarctica [12] and are involved in the dissemination of a large variety of β-lactamases such as cefotaximase (CTX)-M-15 [13], KPC [14,15], VIM [16], OXA-48-like [17-19] or NDM [20]. Recently, reported *K. pneumoniae* ST147 were mainly associated with the production of NDM-1 and NDM-5 carbapenemases in many parts of the world [21-23]. Of note, *K. pneumoniae* ST147 isolates are often considered as hypervirulent since a large proportion has been reported to express known virulence factors such as yersiniabactin or aerobactin [24,25]. Accordingly, *K. pneumoniae* ST147 might become a major threat to public health due to the cumulative effect of the worldwide distribution, high level of antibiotic resistance and increased virulence.

Infections caused by NDM-producers are a severe challenge in healthcare settings, and more studies on appropriate countermeasures are needed. Indeed, the introduction of new β-lactamase inhibitor combinations, such as imipenem-relebactam and meropenem-vaborbactam or ceftazidime-avibactam provided an alternative for treatment of infections caused by Ambler class A and D carbapenemase producers, but the new β-lactamase inhibitors are not effective on MBLs. Cefiderocol, is active against all classes of carbapenemases including MBLs. Unfortunately, recent studies revealed that minimum inhibitory concentrations (MICs) of NDM-producers are significantly higher than of other carbapenemase-producing Enterobacterales [25,26]. Thus, the non-commercially available combination of aztreonam and a β-lactamase inhibitor (avibactam, tazobactam or clavulanic acid) remain the last option to treat infections caused by MBL producers resistant to aztreonam [7,27].

We analysed on the rapid dissemination of NDM-14-producing Enterobacterales in France and investigated their potential origin.

## Methods

### Setting and data collection

Clinical microbiological laboratories of private and public hospitals, nursing homes and communities sent on a voluntary basis Enterobacterales isolates suspected to produce a carbapenemase (usually decreased susceptibility to carbapenems) recovered from clinical and screening specimens to the French National Reference Center for Antibiotic Resistance (NRC) [14]. Following information was accompanied with the isolate: gender and age of patient, specimen type (rectal screen or any type of clinical sample), date of isolation and geographical location of the microbiology laboratory.

### Bacterial isolates

Between June 2014 and June 2022, the French NRC received 431 clinical isolates of *K. pneumoniae* ST147. Of these, 37 were non-duplicate NDM-14-producing isolates cultured from rectal swabs (n = 17), urine samples (n = 13), blood cultures (n = 2), respiratory tract samples (n = 3), an abscess (n = 1) and a superficial wound sample (n = 1). Details on each sample can be seen in Supplementary Table S1.

### Antimicrobial susceptibility testing

Antibiograms (32 molecules) were performed on all isolates of *K. pneumoniae* using disk diffusion method on Mueller-Hinton agar (Biorad, Marnes-la-Coquette, France). Minimal Inhibitory Concentrations (MICs) to imipenem, imipenem-relebactam, meropenem, meropenem-vaborbactam, ceftolozane-tazobactam, piperacillin-tazobactam, aztreonam, cefepime, colistin, amikacin and tobramycin were performed using EUMDROXF Sensititre broth microdilution plates (ThermoFisher Scientific, Dardilly, France) and cefiderocol using UMIC broth microdilution strips (Brucker Daltonics, Bremen, Germany) [28,29].

The MICs of aztreonam-avibactam, aztreonam-tazobactam and aztreonam-clavulanic acid combinations were determined using the Etest strip superposition method as previously described [27].

The results were interpreted according to the European Committee on Antimicrobial Susceptibility Testing (EUCAST) guidelines as updated in 2023 (https://www.eucast.org/fileadmin/src/media/PDFs/EUCAST_files/Breakpoint_tables/v_13.0_Breakpoint_Tables.pdf). In the absence of breakpoints set for aztreonam-inhibitor combinations, the ones for aztreonam were used.

### Transferability of the *bla*_NDM-14_ gene assays

Conjugation experiment was performed as previously described to evaluate the plasmid localisation and the transferability of the *bla*_NDM-14_ gene to other bacterial strains [30]. We used the NDM-14-producing *K. pneumoniae* isolate 310H4 as donor and the azide-resistant *E. coli* J53 as recipient in Luria-Bertani broth using 1:4 donor to recipient ratio. Transconjugants were selected on imipenem (1 µg/mL) and azide (100 µg/mL) supplemented trypticase soy agar plates. Transfer frequency was calculated by dividing the number of transconjugants by the number of donor cells.

### Whole-genome sequencing and phylogenetic analysis

A short-read next-generation sequencing (NGS) was performed on 431 *K. pneumoniae* ST147 isolates (including 37 NDM-14-producing isolates) using a HiSeq system (Illumina Inc, San Diego, United States) (GenBank accession numbers: BioProject PRJNA925084). Illumina reads were assembled using shovill v1.1.0 (https://bio.tools/shovill) and spades v3.14.0. MLST and resistome analysis were performed using PubMLST (https://pubmlst.org/) and Resfinder (https://cge.food.dtu.dk/services/ResFinder/) databases. For phylogenetic single nucleotide polymorphism (SNP) based analysis, NGS sequence reads were mapped to a reference genome included in the study (NDM-14-producing *K. pneumoniae* 310H4) using SNIppy v4.6.0. Metadata and phylogenetic trees were visualised using iTOL v6.5.2 (European Molecular Biology Laboratory). All data resulting from the assembly and SNP analysis are provided in Supplementary Table S2. The virulence score was assessed using Kleborate v2.0.0 (Institut Pasteur, France).

Long-read sequencing was conducted on eight NDM-14-producing *K. pneumoniae* isolates, one per outbreak, and one NDM-1-producing *K. pneumoniae* ST147 isolate phylogenetically close to the NDM-14-producing *K. pneumoniae* ST147 clone. This NDM-1-producing *K. pneumonia*e (312J6) isolate was from a patient hospitalised in Paris. Long-read sequencing was performed using Oxford nanopore MinION technology (Oxford Nanopore, Oxford, United Kingdom) as previously described [31]. By combining data from Illumina and MinION sequencing, we fully assembled and annotated the different plasmids using RAST v2.0 (Rapid Annotation using Subsystem Technology) and CLC Genomics Workbench v.12.0 (Qiagen, Les Ulis, France) software.

### Molecular clock and Bayesian phylogenetic analysis

For molecular clock and Bayesian phylogenetic analysis, only the 37 NDM-14-producing *K. pneumoniae* strains were used. First, the strength of the temporal signal was evaluated using IQTree v1.6.12 and TempEst v1.5.3 softwares (http://tree.bio.ed.ac.uk/software/tempest/) [32,33]. Bayesian analysis was performed using Beast v2.6.1 software (run with a Markov chain Monte Carlo length of 1 × 10^9^, sampling every 5 × 10^3^ steps) as previously described [34]. We used model parameters that had the best fit: generalised time reversible (GTR) substitution model, lognormal relaxed clock and constant population size as previously used with a *K. pneumoniae* ST147 population [35]. Parameter estimates were visualised using Tracer v1.7.2 (https://beast.community/tracer), a maximum clade credibility tree was obtained with TreeAnnotator V2.6.0 (https://beast2.blogs.auckland.ac.nz/treeannotator/) and visualised and annotated with FigTree V1.4.4 (http://tree.bio.ed.ac.uk/software/figtree/). To compare the evolution rate of NDM-14-producing *K. pneumoniae* ST147, we also performed this analysis on 100 *K. pneumoniae* ST147 isolates producing another type of carbapenemase recovered from the French NRC collection.

## Results

### Rapid emergence of NDM-14-producing *Klebsiella pneumoniae* ST147

**Figure 1:**
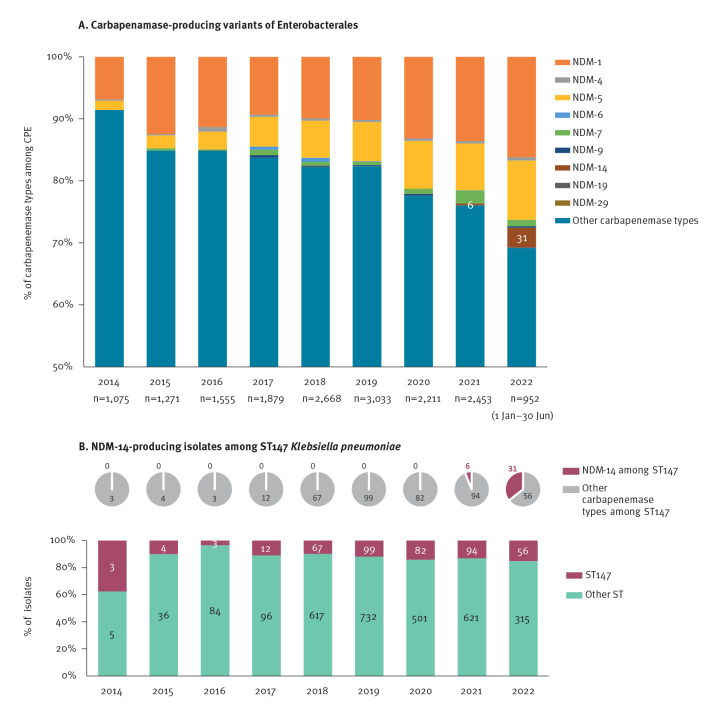
Distribution and number of isolates of carbapenemase-producing Enterobacterales, including NDM-14-producing *Klebsiella pneumoniae,* received at the French National Reference Center for Antibiotic Resistance, 1 January 2014–30 June 2022 (n = 17,097)

CPE: carbapenemase-producing Enterobacterales; NDM: New Delhi metallo-β-lactamase; ST: sequence type.

All 37 NDM-14-producing isolates received at the French NRC were *K. pneumoniae* ST147. The proportion of NDM-producing Enterobacterales of all CPEs received at the French NRC increased from 92/1,075 (8.6%) in 2014 to 587/2,453 (23.9%) in 2021. In 2022, between 1 January and 30 June, 293 NDM-producing Enterobacterales representing 30.8% of all 952 CPEs were received (Figure 1A). Among the NDM-producers, NDM-1 and NDM-5 were the most common variants each year (Figure 1A). No NDM-14-producers were received at the French NRC before 2021. However, in 2021, 6 (1%) of 587 and in 2022 (January–June), 31 (10.6%) of the 293 NDM-producing isolates were NDM-14 (Figure 1B). More information can be seen in Supplementary Table S1.

The total number of *K. pneumoniae* ST147 isolates received at the French NRC also increased since 2014 (three in 2014, 94 in 2021 and 56 between1 January and 30 June 2022) (Figure 1B). These *K. pneumoniae* ST147 isolates were associated with diverse carbapenemases. However, since 2021, the most common carbapenemase among *K. pneumoniae* ST147 isolates has been NDM-14, reaching 41.3% in 2022 (Figure 1B).

### Outbreaks involving a unique cluster of NDM-14-producing *Klebsiella pneumoniae* ST147 linked to Morocco

**Figure 2:**
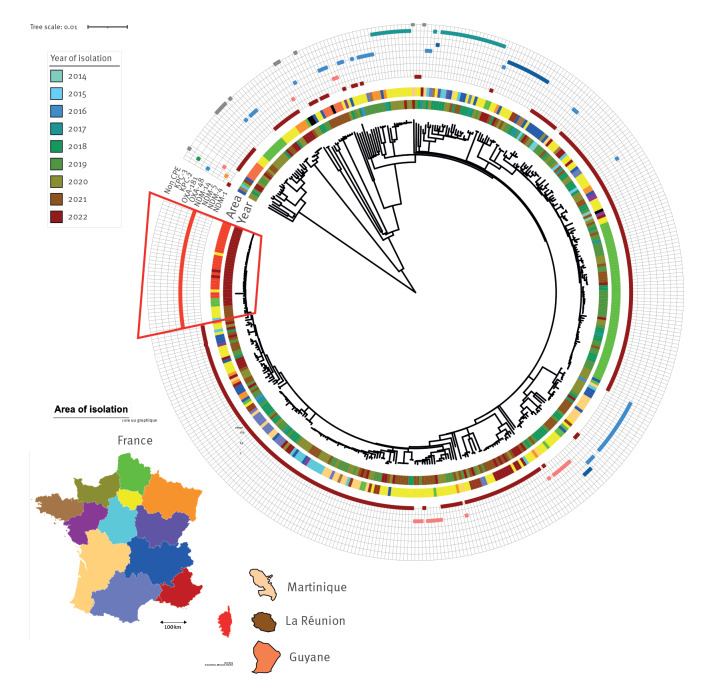
Phylogenetic tree of carbapenemase-producing *Klebsiella pneumoniae* ST147 isolates received at the French National Reference Center for Antibiotic Resistance, France, 1 January 2014–30 June 2022 (n = 431)

We performed short-read next-generation sequencing on all non-duplicate *K. pneumoniae* ST147 received at the French NRC between 2014 and June 2022 (n = 431). More details on the isolates can be seen in Supplementary Table S1. Phylogenetic analysis revealed a wide diversity with multiple subclades, distributed in different regions of France (Figure 2).

ST: sequence type.

The red box corresponds to NDM-14 producing *Klebsiella pneumoniae.*

Among all 431 carbapenem-resistant *K. pneumoniae* ST147, several carbapenemases were identified: NDM-1 (n = 254, 58.9%), OXA-48 (n = 42, 9.7%), NDM-14 (n = 37, 8.6%), KPC-3 (n = 30, 7.0%), OXA-181 (n = 17, 3.9%), NDM-5 (n = 13, 3%), NDM-1 and OXA-48 (n = 17, 3.9%), NDM-5 and OXA-181 (n = 8, 1.9%), OXA-232 (n = 1, 0.2%), NDM-6 (n = 1, 0.2%) and NDM-4 and KPC-2 (n = 1, 0.2%). Noticeably, 10 isolates (2.3%) did not produce any carbapenemase (Figure 2). More details can be seen in Supplementary Table S1.

**Figure 3:**
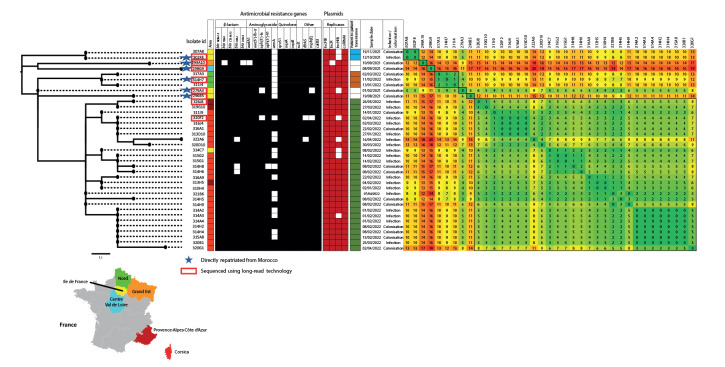
Phylogenetic tree and a heatmap of NDM-14-producing *Klebsiella pneumoniae* ST147 isolates received at the French National Reference Center for Antibiotic Resistance, France, 1 January 2014–30 June 2022 (n = 37)

NDM: New Delhi metallo-β-lactamase; SNP: single nucleotide polymorphism; ST: sequence type.

Some carbapenemases were distributed in many subclades (such as NDM-1, NDM-5, OXA-48), whereas others were associated with a limited number of subclades (e.g. OXA-181, KPC-3). However, the 37 NDM-14 producers were part of a single subclade that emerged recently (first isolation in France in February 2021) with a national distribution: Corsica (n = 24), Île-de-France (n = 5), Nord (n = 3), Provence-Alpes-Côte d’Azur (n = 3), Grand Est (n = 1) and Centre-Val de Loire (n = 1) (Figure 2 and Figure 3).

Blue star: patients repatriated from Morocco; red box: bacterial isolates sequenced using long-read sequencing (MinION).

Antimicrobial resistance: black indicates a resistance gene was detected, white the resistance gene not detected.

Plasmids: red indicates detection of a plasmid, white plasmid not detected.

Patient-to-patient contact: the isolates belonging to the same cluster are indicated with blue (n = 2), brown (n = 3) or green (n = 28).

Numbers in the boxes of the heatmap correspond to the number of SNPs between the isolates.

A phylogenetic tree and a SNP matrix were built with all NDM-14-producing *K. pneumoniae* to observe the diversity within this population (Figure 3). All NDM-14-producing *K. pneumoniae* were relatively close with a maximum number of 20 SNPs between two isolates as can be seen in Supplementary Figure S1. The SNP matrix analysis confirmed by epidemiological investigations highlighted three outbreaks with proven patient-to-patient transmissions: one in Île-de-France involving two patients, one in Nord involving three patients and the largest occurring in Corsica with 26 patients. Regarding the outbreak mostly occurring in Corsica (28 isolates from 26 patients), all the NDM-14-producing *K. pneumoniae* were isolated from patients hospitalised in one hospital of the island and then transferred to several other clinical facilities or discharged at home. More information can be seen in Supplementary Figure S2. Furthermore, three isolates from Provence-Alpes-Côte d’Azur and one from Île-de-France were carried by patients previously hospitalised in Corsica. In most cases (n=6), the index patient was directly repatriated from Morocco, from several hospitals in Casablanca region (Figure 3).

Of note, 34/37 (91.9%) of the NDM-14-producing *K. pneumoniae* ST147 isolated in France possessed a high virulence score of 4/5 according to Kleborate classification with expression of two main virulence factors, the yersiniabactin and the aerobactin. More details can be seen in Supplementary Figure S5.

### Plasmid analysis of NDM-14-producing *Klebsiella pneumoniae* ST147

Several replicases were identified in the collection of NDM-14 producing strains. Plasmids IncFIB and ColRNAI were observed in all 37 strains, IncR in 36 strains and IncHIB in 29 strains (Figure 3).

**Figure 4:**
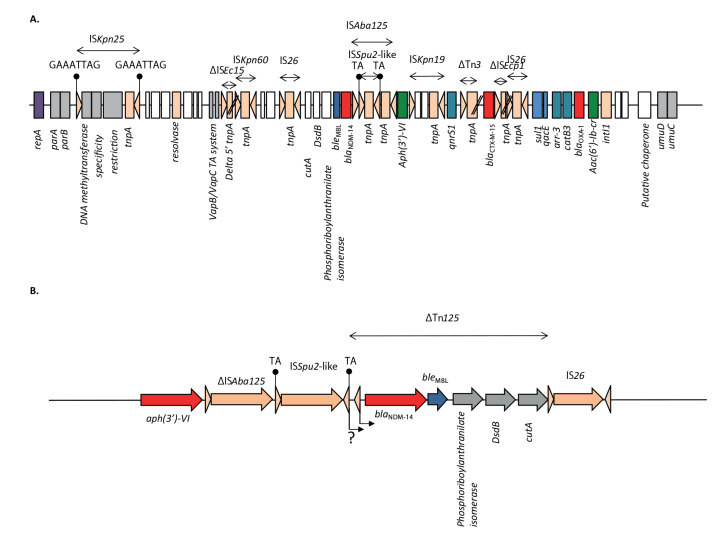
Schematic representation of the *bla*_NDM-14_-carrying plasmid (A) and of the close genetic environment of *bla*_NDM-14_ (B)

The *bla*_NDM-14_-carrying plasmid has been entirely reconstructed using long-read sequencing on eight NDM-14-producing *K. pneumoniae* isolates (Figure 4). All strains harboured an IncFIB plasmid of 54,061 base pairs (bp) in size (GenBank accession number PRJNA925431). Analysis of the close genetic context of *bla*_NDM-14_ revealed the presence of a IS*Aba125* likely involved in the carbapenemase expression [36]. By contrast to what is commonly reported in NDM-producing Enterobacterales, this IS*Aba125* was truncated by IS*Spu-2* (Figure 4). Upstream of IS*Aba125*, a copy of *aph*(3’)-VI was identified, frequently associated with *bla*_NDM-1_ in *Acinetobacter* species [37]. Downstream of *bla*_NDM-14_, a remnant of Tn*125,* truncated by a copy of IS*26,* was evidenced [38]. Resistome analysis also revealed the presence of the *bla*_CTX-M-15_ ESBL encoding gene associated to IS*Ecp1*. Remaining resistance genes were identified embedded in a class 1 integron carrying *aac(6’)-Ib-cr*, *bla*_OXA-1_, *catB3*, *arr-3* and *sul1*.

Panel A and B: replication initiation protein is indicated by a purple box, β-lactamases by red boxes, aminoglycoside-modifying enzymes by green boxes, other resistance genes by blue boxes and other functions by grey boxes. Hypothetical proteins are indicated by white boxes. Insertion sequences and other mobile elements are indicated by orange boxes whereas inverted repeats are indicated by orange triangles. Direct repeats are represented by vertical lines and the sequences is indicated.

Panel B: orientations of coding sequences are indicated.

Replication and partitioning systems of the *bla*_NDM-14_-carrying IncFIB plasmid were identical to *bla*_KPC_-carrying pKpQIL-like plasmids [39]. However, this plasmid did not possess any conjugative transfer system (Figure 4). Surprisingly, the *bla*_NDM-14_ gene was successfully transferred into *E. coli* J53 by conjugation with a transfer frequency of 6.5 (± 2.5) × 10^− 7^ transconjugant/donor. *Escherichia coli* J53 transconjugants carrying *bla*_NDM-14_ exhibited resistance to all tested β-lactams including aztreonam. Whole genome sequencing performed on one transconjugant revealed the presence two replicases of IncFIB-type and of IncH1B-type. The IncH1B-type plasmid was of ca 350 kilobases (kb) in size and was closely related to pVIR147Tu.12PI (GenBank Accession Number CP072918). It possesses a complete transfer operon as well as aerobactin and tellurite resistance operons. This IncH1B-type plasmid probably behaves as a helper plasmid allowing conjugation of the *bla*_NDM-14_ carrying plasmid.

A long-read sequencing was also performed on the isolate 312J6, a NDM-1 producing *K. pneumoniae* ST147 that is very close to the cluster of NDM-14 producers (30 to 36 SNPs). This strain also harboured IncFIB, IncR, IncHIB and ColRNAI type plasmids, as well as the same antimicrobial resistance genes (*bla*_CTX-M-15_, *bla*_OXA-1_, *bla*_OXA-9_, *bla*_TEM-1_, *aac(6’)-Ib-cr, qnrS1, oqxA, oqxB, sul1, dfrA5, mph(E) or catB3*), except that *bla*_NDM-14_ was replaced by *bla*_NDM-1_. Comparison of both IncFIB-type plasmid sequences revealed only one SNP of difference in *bla*_NDM-1_. This result indicated that the *bla*_NDM-14_ carrying plasmid derived directly from the plasmid carrying *bla*_NDM-1_. The BLAST analysis of the complete *bla*_NDM-14_-carrying plasmid identified similar plasmids in database (100% coverage with 99% identity). However, all submitted plasmids carried *bla*_NDM-1_ gene and seem to have disseminate in Italy (GenBank accession number CP074090), in Thailand (LC521845) and in Switzerland (CP082992).

### High level of antimicrobial resistance of NDM-14-producing *Klebsiella pneumoniae* ST147

All 37 isolates were highly resistant to imipenem, meropenem, imipenem-relebactam, meropenem-vaborbactam, ceftolozane-tazobactam, ceftadizime-avibactam, piperacillin-tazobactam, aztreonam, cefepime, amikacin, and tobramycin (Table, Figure 3). All isolates were susceptible to cefiderocol but with MICs on the clinical breakpoint of 2 mg/L. Susceptibility to colistin was 94.6%. In addition, all isolates were susceptible to the combination aztreonam-avibactam with low MICs (MIC_50_ = 0.25 mg/L, MIC_90_ = 0.38 mg/L) and 91.9% to the combination aztreonam-clavulanic acid (MIC_50_ = 0.5 mg/L, MIC_90_ = 1.5 mg/L). These isolates had high MICs for the aztreonam-tazobactam combination (MIC_50_ = 128 mg/L, MIC_90_ = 256 mg/L) (Table). According to disk diffusion method, all isolates were also resistant to other families of antimicrobials such as chloramphenicol, quinolones or sulfonamides and most of them harboured additional acquired resistance determinants such as, *aac(6’)-Ib-cr, qnrS1, sul1, dfrA5, mph(E) or catB3* (Figure 3).

**Table:** Minimum inhibitory concentrations of antimicrobials on NDM-14-producing isolates of *Klebsiella pneumoniae*, France (n = 37)

Characteristics	Antimicrobial															
	Imipenem	Imipenem-relebactam	Meropenem	Meropenem-vaborbactam	Ceftolozane-tazobactam	Cefiderocol	Ceftazidime-avibactam	Piperacillin-tazobactam	Aztreonam	Cefepime	Colistin	Amikacin	Tobramycin	Aztreonam-avibactam	Aztreonam-clavulanic acid	Aztreonam-tazobactam
Isolate id	Minimum inhibitory concentrations mg/L^a^															
279 A3	> 8	> 8	> 16	> 16	> 8	**2**	> 16	> 32	> 32	> 16	≤ **0.5**	> 32	> 4	**0.25**	**0.5**	256
296 B5	> 8	> 8	> 16	> 16	> 8	**2**	> 16	> 32	> 32	> 16	≤ **0.5**	> 32	> 4	**0.38**	**0.5**	128
298 G9	> 8	> 8	> 16	> 16	> 8	**2**	> 16	> 32	> 32	> 16	≤ **0.5**	> 32	> 4	**0.25**	**0.5**	> 256
299 A10	> 8	> 8	> 16	> 16	> 8	**2**	> 16	> 32	> 32	> 16	≤ **0.5**	> 32	> 4	**0.25**	**0.38**	96
302 F8	> 8	> 8	> 16	> 16	> 8	**2**	> 16	> 32	> 32	> 16	32	> 32	> 4	**0.25**	**0.5**	96
307 A8	> 8	> 8	> 16	> 16	> 8	**2**	> 16	> 32	> 32	> 16	32	> 32	> 4	**0.25**	**0.5**	96
310 H4	> 8	> 8	> 16	> 16	> 8	**2**	> 16	> 32	> 32	> 16	≤ **0.5**	> 32	> 4	**0.25**	**0.5**	192
311 I4	> 8	> 8	> 16	> 16	> 8	**2**	> 16	> 32	> 32	> 16	≤ **0.5**	16	> 4	**0.25**	**0.5**	96
311 I9	> 8	> 8	> 16	> 16	> 8	**2**	> 16	> 32	> 32	> 16	≤ **0.5**	> 32	> 4	**0.25**	**0.5**	96
313 D10	> 8	> 8	> 16	> 16	> 8	**2**	> 16	> 32	> 32	> 16	≤ **0.5**	> 32	> 4	**0.38**	**0.5**	192
313 H5	> 8	> 8	> 16	> 16	> 8	**2**	> 16	> 32	> 32	> 16	≤ **0.5**	> 32	> 4	**0.25**	**0.5**	192
314 A2	> 8	> 8	> 16	> 16	> 8	**2**	> 16	> 32	> 32	> 16	≤ **0.5**	> 32	> 4	**0.25**	**0.5**	192
314 A3	> 8	> 8	> 16	> 16	> 8	**2**	> 16	> 32	> 32	> 16	≤ **0.5**	> 32	> 4	**0.25**	**0.5**	192
314 A4	> 8	> 8	> 16	> 16	> 8	**2**	> 16	> 32	> 32	> 16	≤ **0.5**	> 32	> 4	**0.25**	**0.5**	192
314 C7	> 8	> 8	> 16	> 16	> 8	**2**	> 16	> 32	> 32	> 16	≤ **0.5**	> 32	> 4	**0.38**	*1.5*	> 256
314 H2	> 8	> 8	> 16	> 16	> 8	**2**	> 16	> 32	> 32	> 16	≤ **0.5**	> 32	> 4	**0.25**	*2*	> 256
314 H4	> 8	> 8	> 16	> 16	> 8	**2**	> 16	> 32	> 32	> 16	≤ **0.5**	> 32	> 4	**0.25**	**0.5**	192
314 H5	> 8	> 8	> 16	> 16	> 8	**2**	> 16	> 32	> 32	> 16	≤ **0.5**	> 32	> 4	**0.25**	*2*	> 256
314 H6	> 8	8	> 16	> 16	> 8	**2**	> 16	> 32	> 32	> 16	≤ **0.5**	> 32	> 4	**0.25**	**0.5**	192
314 H7	> 8	> 8	> 16	> 16	> 8	**2**	> 16	> 32	> 32	> 16	≤ **0.5**	> 32	> 4	**0.25**	**0.5**	192
314 H8	> 8	> 8	> 16	> 16	> 8	**2**	> 16	> 32	> 32	> 16	≤ **0.5**	> 32	> 4	**0.25**	*1.5*	128
314 H9	> 8	> 8	> 16	> 16	> 8	**2**	> 16	> 32	> 32	> 16	≤ **0.5**	> 32	> 4	**0.25**	**0.5**	128
315 A8	> 8	> 8	> 16	> 16	> 8	**2**	> 16	> 32	> 32	> 16	≤ **0.5**	> 32	> 4	**0.38**	**0.5**	192
315 G1	8	> 8	> 16	> 16	> 8	**2**	> 16	> 32	> 32	> 16	≤ **0.5**	> 32	> 4	**0.25**	*2*	192
315 G2	> 8	> 8	> 16	> 16	> 8	4	> 16	> 32	> 32	> 16	≤ **0.5**	> 32	> 4	**0.25**	**0.5**	192
316 A1	> 8	> 8	> 16	> 16	> 8	**2**	> 16	> 32	> 32	> 16	≤ **0.5**	> 32	> 4	**0.25**	**0.5**	192
316 A9	> 8	> 8	> 16	> 16	> 8	**2**	> 16	> 32	> 32	> 16	≤ **0.5**	> 32	> 4	**0.25**	**0.5**	96
316 J4	> 8	> 8	> 16	> 16	> 8	**2**	> 16	> 32	> 32	> 16	≤ **0.5**	> 32	> 4	**0.25**	**0.5**	96
317 A3	> 8	> 8	> 16	> 16	> 8	**2**	> 16	> 32	> 32	> 16	≤ **0.5**	> 32	> 4	**0.38**	**0.5**	96
319 G10	> 8	> 8	> 16	> 16	> 8	**2**	> 16	> 32	> 32	> 16	≤ **0.5**	> 32	> 4	**0.38**	**0.5**	96
320 B1	> 8	> 8	> 16	> 16	> 8	**2**	> 16	> 32	> 32	> 16	≤ **0.5**	> 32	> 4	**0.38**	**0.5**	128
320 D10	> 8	> 8	> 16	> 16	> 8	**2**	> 16	> 32	> 32	> 16	≤ **0.5**	> 32	> 4	**0.25**	**0.5**	128
320 F2	> 8	> 8	> 16	> 16	> 8	**2**	> 16	> 32	> 32	> 16	≤ **0.5**	16	> 4	**0.25**	**0.5**	96
320 G1	> 8	> 8	> 16	> 16	> 8	**2**	> 16	> 32	> 32	> 16	≤ **0.5**	> 32	> 4	**0.25**	**0.38**	96
321 B6	> 8	> 8	> 16	> 16	> 8	**2**	> 16	> 32	> 32	> 16	≤ **0.5**	> 32	> 4	**0.25**	**0.38**	96
322 A6	> 8	> 8	> 16	> 16	> 8	**2**	> 16	> 32	> 32	> 16	≤ **0.5**	> 32	> 4	**0.25**	**0.38**	96
326 J8	> 8	> 8	> 16	> 16	> 8	**2**	> 16	> 32	> 32	> 16	≤ **0.5**	> 32	> 4	**0.25**	**0.38**	96
Determination of intrinsic activity																
MIC_50_	> 8	> 8	> 16	> 16	> 8	2	> 16	> 32	> 32	> 16	≤ 0.5	> 32	> 4	0.25	0.5	128
MIC_90_	> 8	> 8	> 16	> 16	> 8	2	> 16	> 32	> 32	> 16	≤ 0.5	> 32	> 4	0.38	1.5	256
Percentage of susceptible isolates																
Total	0%	0%	0%	0%	0%	100%	0%	0%	0%	0%	94.6%	0%	0%	100%	91.9%	0%

Id: identification; MIC: minimum inhibitory concentration; NDM: New Delhi metallo-β-lactamase.

Isolates classified susceptible are indicated in bold and those intermediate in italics.

MIC_50_ and MIC_90_ are defined as the minimum inhibitory concentration of 50% and 90% of tested isolates.

### Dating appearance and evolution rate of NDM-14-producing *Klebsiella pneumoniae* ST147 cluster

Using BEAST analysis, we detected a temporal signal in the NDM-14-producing *K. pneumoniae* (R^2^ = 0.3236), that made possible the estimation of the evolutionary rate and the dating of emergence of the NDM-14-producing *K. pneumoniae* ST147 cluster. More details can be seen in Supplementary Figure S3. Bayesian phylogenetic analysis was performed on all NDM-14-producing *K. pneumoniae*, the evolutionary rate was estimated at 1.11 × 10^− 6^ substitutions per site and per year (95% highest posterior density (HPD), 8.67 × 10^− 7^–1.61 × 10^− 6^), corresponding to 5.61 SNPs per genome per year (4.38–8.13 SNPs). The evolutionary rate of other *K. pneumoniae* ST147 isolates was close to that of NDM-14: an estimation of 1.30 × 10^−6^ substitutions per site and per year (95% HPD, 7.67 × 10^− 7^–1.90 × 10^− 6^) (6.2 SNPs per year, range 3.65–9.05 SNPs). This analysis estimated that the NDM-14-producing *K. pneumoniae* ST147 clone appeared in 2020.3 (i.e. April 2020) (95% confidence interval (CI): 2019.5–2021.2, i.e. June 2019–February 2021) likely deriving from a NDM-1-producing *K. pneumoniae* ST147*.* More information is available in Supplementary Figure S4.

## Discussion

To our knowledge, this is the first description of NDM-14-producing Enterobacterales. Previously, one isolate of NDM-14-producing *A. lwoffii* has been described [9]. Furthermore, since 2021, the French NRC has received 37 isolates of NDM-14-producing *K. pneumoniae*, obtained from outbreaks in various regions of France. The NDM-14 variant, rapidly disseminated in France, represented 10.6% of NDM-producing Enterobacterales in 2022. The MLST analyses we performed revealed that all NDM-14-producing *K. pneumoniae* belonged to ST147. In addition, SNPs analysis demonstrated that the emergence and the rapid dissemination of NDM-14-producing *K. pneumoniae* ST147 seemed linked to a single clone. Indeed, the maximum number of SNPs between two isolates was of 20 SNPs, which is under the cut-off of 21 SNPs set to separate two clones in previously studied ST258/512 *K. pneumoniae* outbreaks [40]. Furthermore, epidemiological investigations combined with genomic analysis enabled to identify several independent outbreaks. Accordingly, this cut-off of 21 SNPs that has been set to decipher clonality in ST258/512 *K. pneumoniae* has to be used with caution when other STs are studied. These results highlight the importance of combining epidemiological investigation and whole genome comparison to analyse outbreak data. Although, no published data on the distribution of this NDM-14-producing *K. pneumoniae* clone in Morocco are available, most of the index patients were directly repatriated from this country, suggesting a dissemination of this clone in hospitals of the Casablanca Region.

The conjugation assays demonstrated the transferability of the *bla*_NDM-14_-carrying plasmid only in the presence of another conjugative plasmid (helper). Plasmid sequencing confirmed the absence of a conjugative transfer system on the plasmid harbouring *bla*_NDM-14_. Long-read sequencing enabled to obtain the complete sequence of the 54,061 bp plasmid of IncF1B type carrying *bla*_NDM-14_. This plasmid was very different from the *bla*_NDM-14_-carrying plasmid reported from *A. lwoffii* in China [9] and had strong homology with the *bla*_NDM-1_ carrying plasmid of *K. pneumoniae* 312J6, with only one mutation in the *bla*_NDM_ gene. These findings suggest that the presence of the *bla*_NDM-14_ gene in *K. pneumoniae* ST147 is more likely related to a mutation in the *bla*_NDM-1_ gene already present in a *K. pneumoniae* ST147 strain rather than to a mobilisation of the *bla*_NDM-14-_carrying plasmid present in *A. lwoffii*.

In our study, the mean evolutionary rate for all *K. pneumoniae* ST147 was estimated at 1.30 x 10^− 6^ substitution/site/year (95% HPD; 7.67 × 10^− 7^–1.90 x 10^− 6^; 6.2 SNPs per year, range 3.65–9.05 SNPs). This evolutionary rate is consistent with the one calculated in another study on *K. pneumoniae* ST147 (1.45 × 10^− 6^ substitution/site/year) [35]. Furthermore, this evolution rate is similar to those observed in other high-risk clones involved in the dissemination of resistances genes such as ST258 (1.03 × 10^− 6^ substitutions/site/year) and ST307 (1.18 × 10^− 6^ substitutions/site/year) [41,42]. However, the rate remained smaller than the one calculated for ST101 (2.85 × 10^− 6^ substitutions/site/year) [43]. Our analysis also indicated that NDM-14-producing *K. pneumoniae* ST147 emerged in April 2020, thus confirming the recent emergence of this rapidly disseminated clone.

As often observed for aztreonam resistant metallo-β-lactamase producers, NDM-14-producing *K. pneumoniae* are highly resistant to most antibiotics. Cefiderocol remained active against most of these strains but with MICs very close to the clinical breakpoint, highlighting hypothetical difficulties of successful treatments with this last resort molecule. As previously demonstrated, the combination aztreonam-avibactam or aztreonam-clavulanic acid remains the antimicrobial with the lowest MICs on NDM-producing bacteria and may represent the best therapeutic options [27].

## Conclusion

The recent and rapid emergence of NDM-14-producing *K. pneumoniae* ST147 isolated from patients in France directly repatriated from Morocco suggests a spread of this clone particularly in the Casablanca region. This clone likely emerged recently from an endemic NDM-1-producing *K. pneumoniae* ST147.

## Supplementary Data

2179000 Supplementary Material
