# Supplementary material for: Emergence and rapid dissemination of highly resistant NDM-14-producing Klebsiella pneumoniae ST147, France, 2022
Source: Euro Surveill. 2023 Oct 19;28(42):2300095. doi: 10.2807/1560-7917.ES.2023.28.42.2300095 (PMC10588306; doi:10.2807/1560-7917.ES.2023.28.42.2300095)
Supplement: Supplementary Material [file 23-00095_DORTET_Supplement.pdf]

This supplementary material is hosted by Eurosurveillance as supporting information alongside the article Emergence and rapid dissemination of highly resistant NDM-14-producing *Klebsiella pneumoniae* of ST-147 in France, on behalf of the authors, who remain responsible for the accuracy and appropriateness of the content. The same standards for ethics, copyright, attributions and permissions as for the article apply. Supplements are not edited by *Eurosurveillance* and the journal is not responsible for the maintenance of any links or email addresses provided therein.

SUPPLEMENTARY DATA

Figure S1: SNP matrix (Single-Nucleotide Polymorphism) for the 37 NDM-14-producing *Klebsiella pneumoniae* ST-147 received at the French National Reference Center for Carbapenem-resistant Enterobacterales from 1st January 2014 to 30th June 2022. Blue star: Patients repatriated from Morocco

Figure S2: Synoptic curve of patients infected or colonized with ST-147 NDM-14-producing *Klebsiella pneumoniae* responsible for the outbreak in Corsica

Figure S3: Temporal signal analysed with Iq-Tree and TempEst softwares. Correlation between the genetic distance of each sequence to the NDM-14 producing *K. pneumoniae* strains SNPs phylogeny and the date of isolation.

Figure S4: Time-scaled Bayesian phylogeny of 37 ST-147 NDM-14-producing *K. pneumoniae*. The 3 outbreaks described in this study are highlighted with different colours.

Figure S5: Virulence scores established with Kleborate for the 431 carbapenem-resistant *Klebsiella pneumoniae* ST-147 received at the French National Reference Center for Carbapenem-resistant Enterobacterales from 1st January 2014 to 30th June 2022. NDM-14 producing isolates are indicated with a purple circle. The virulence score ranges from 0 to 5 : **0** = negative for all of yersiniabactin (*ybt*), colibactin (*clb*), aerobactin (*iuc*); **1** = yersiniabactin only; **2** = yersiniabactin and colibactin (or colibactin only); **3** = aerobactin (without yersiniabactin or colibactin); **4** = aerobactin with yersiniabactin (without colibactin); **5** = yersiniabactin, colibactin and aerobactin

Table S1: Global characteristics of all isolates included in the study

Table S2: Genome assembly quality metrics of the 37 NDM-14-producing *K. pneumoniae* ST-147 used in the study.

Figure S1: SNP matrix (Single-Nucleotide Polymorphism) for the 37 NDM-14-producing *Klebsiella pneumoniae* ST-147 received at the French National Reference Center for Carbapenem-resistant Enterobacterales from 1st January 2014 to 30th June 2022. Blue star: Patients repatriated from Morocco

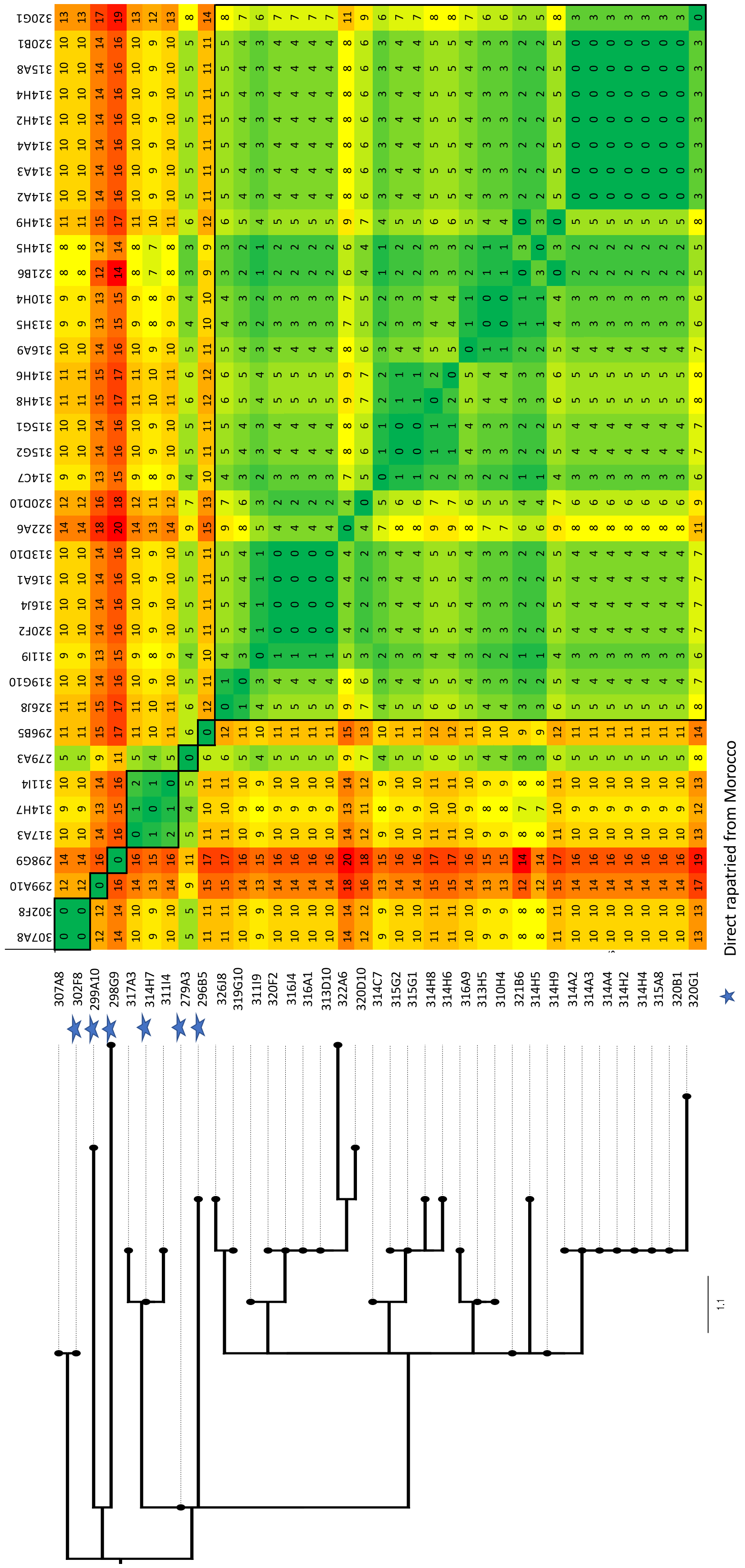

Figure S2: Synoptic curve of patients infected or colonized with ST-147 NDM-14-producing *Klebsiella pneumoniae* responsible for the outbreak in Corsica

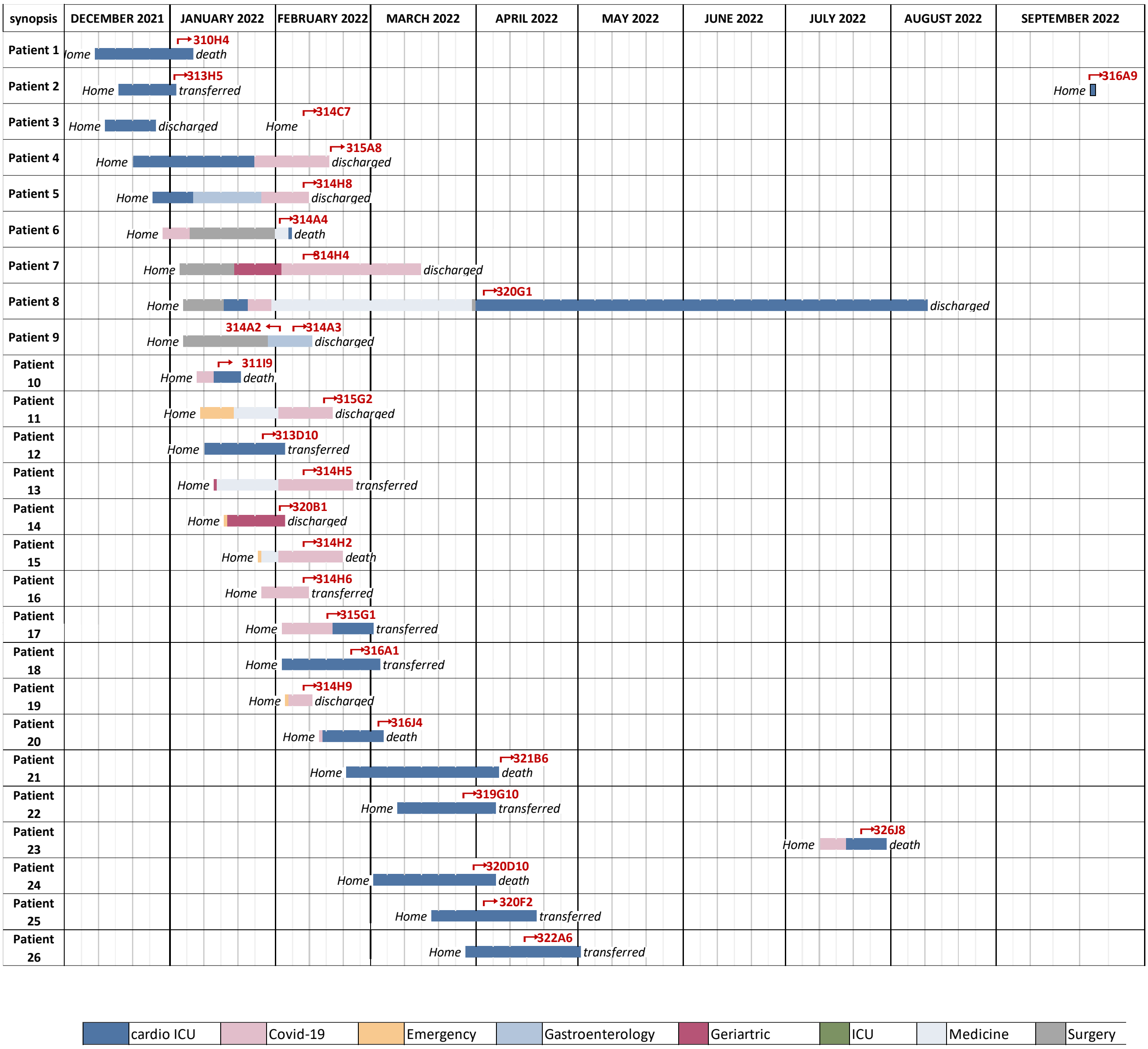

Figure S3: Temporal signal analysed with Iq-Tree and TempEst softwares. Correlation between the genetic distance of each sequence to the NDM-14 producing *K. pneumoniae* strains SNPs phylogeny and the date of isolation.

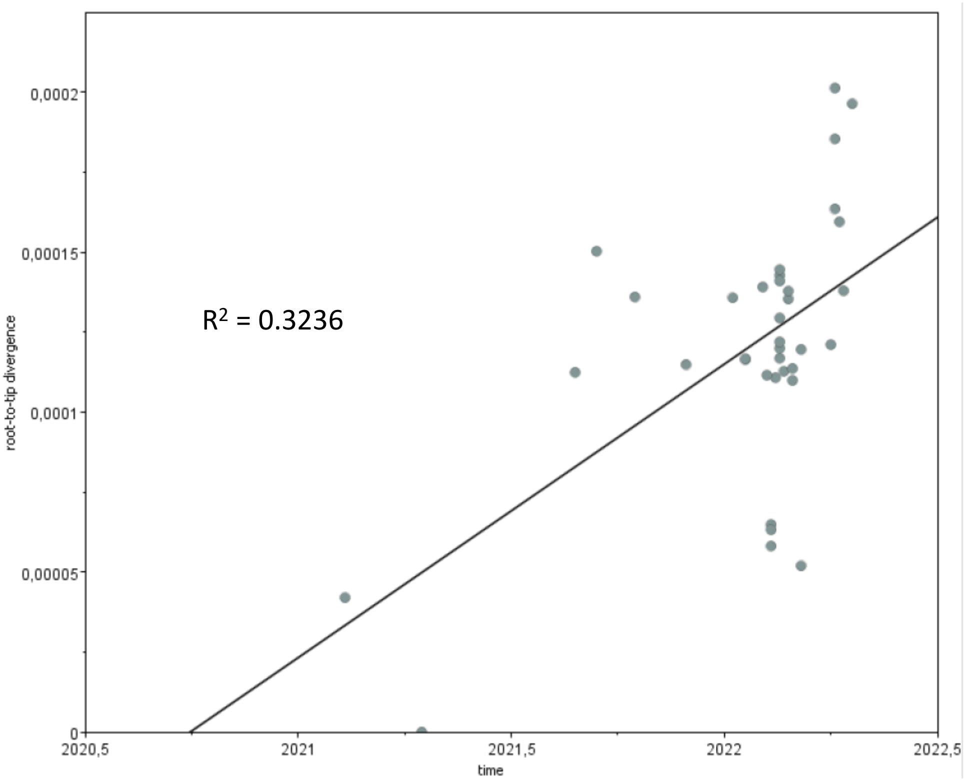

Figure S4: Time-scaled Bayesian phylogeny of 37 ST-147 NDM-14-producing *K. pneumoniae*. The 3 outbreaks described in this study are highlighted with different colours.

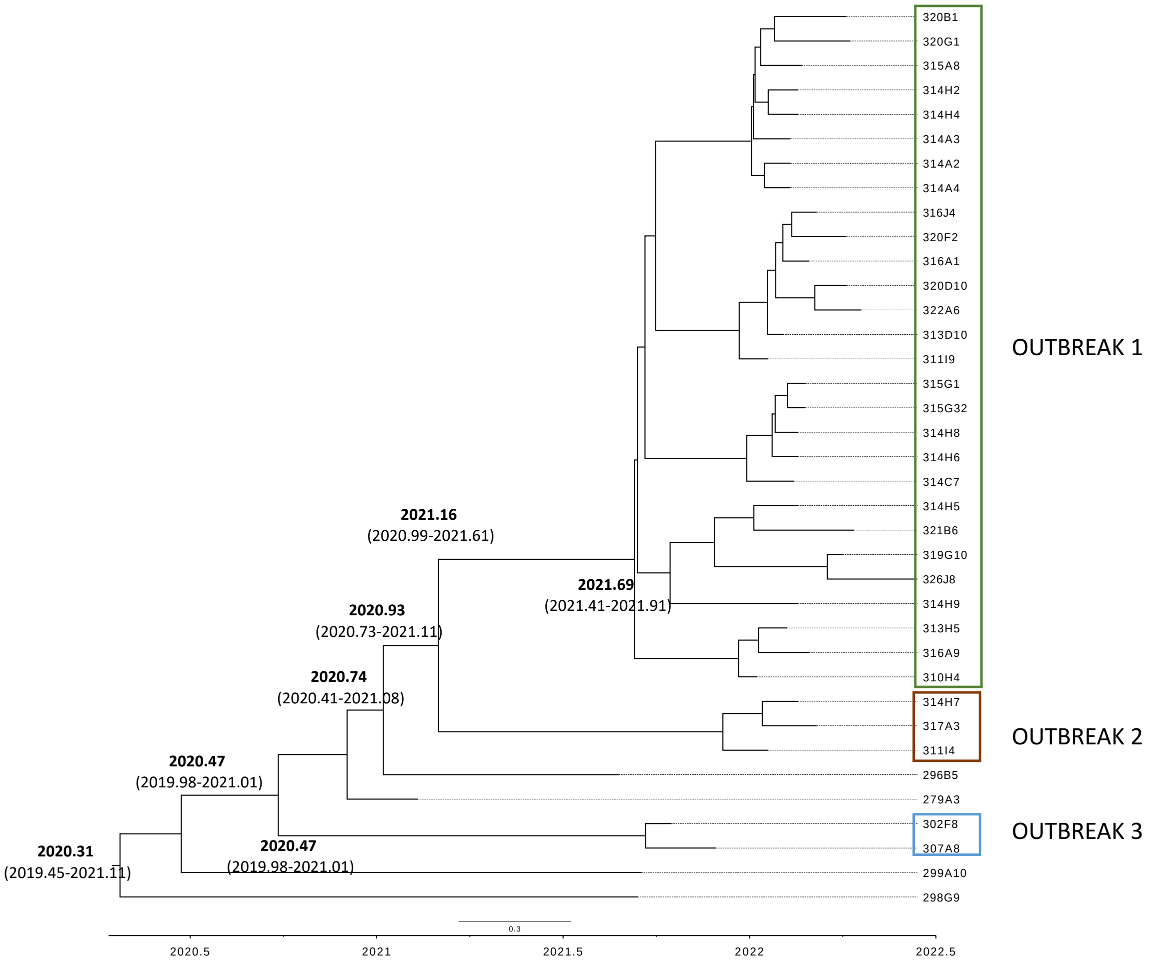

Figure S5: Virulence scores established with Kleborate for the 431 carbapenem-resistant *Klebsiella pneumoniae* ST-147 received at the French National Reference Center for Carbapenem-resistant Enterobacterales from 1st January 2014 to 30th June 2022. NDM-14 producing isolates are indicated with a purple circle. The virulence score ranges from 0 to 5 : **0** = negative for all of yersiniabactin (*ybt*), colibactin (*clb*), aerobactin (*iuc*); **1** = yersiniabactin only; **2** = yersiniabactin and colibactin (or colibactin only); **3** = aerobactin (without yersiniabactin or colibactin); **4** = aerobactin with yersiniabactin (without colibactin); **5** = yersiniabactin, colibactin and aerobactin

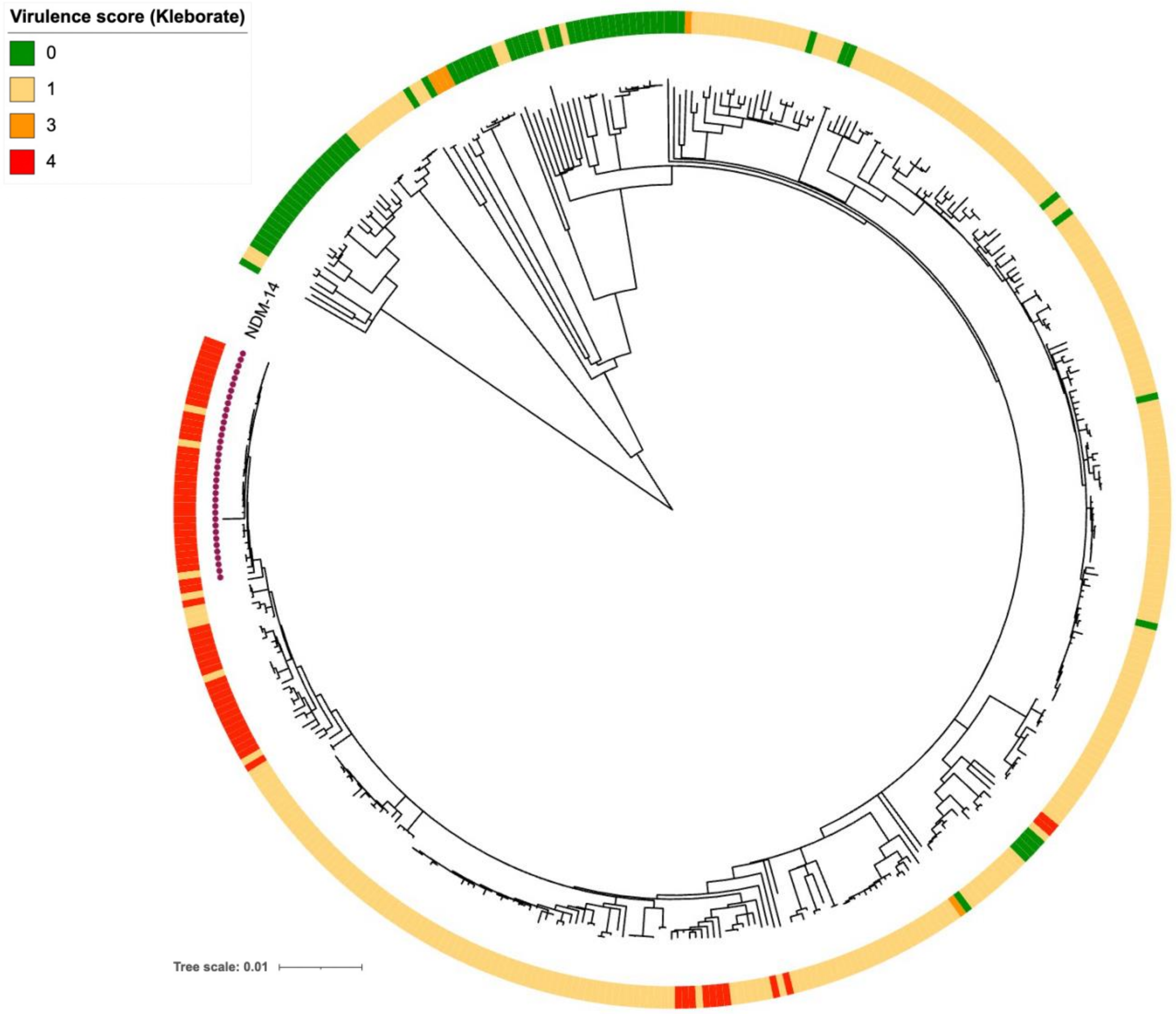

Table S1: Global characteristics of all isolates included in the study

Strain	Date of isolation	Specimen	Region of isolation	Carbapenemase type	Gender	Age
86D6	17/08/2015	Rectal swab	Ile-de-France	NDM-1	M	57
86I2	21/08/2015	Rectal swab	Auvergne-Rhône-Alpes	NDM-1	M	67
93D2	27/10/2015	Rectal swab	Ile-de-France	NDM-1	F	84
59E6	08/09/2014	Not determined	Pays de la Loire	NDM-1	M	75
67C8	27/11/2014	Not determined	Normandie	NDM-1	F	43
102F3	15/02/2016	Rectal swab	Ile-de-France	NDM-1	M	79
106E10	08/04/2016	Blood culture	Nouvelle-Aquitaine	OXA-48	F	76
110G7	06/06/2016	Rectal swab	Ile-de-France	NDM-1	M	28
132J10	01/03/2017	Rectal swab	Grand Est	KPC-3	F	85
132J3	28/02/2017	Rectal swab	Bretagne	OXA-181	M	79
133I5	09/03/2017	Rectal swab	Bretagne	OXA-181	M	68
136A6	03/04/2017	Rectal swab	La Réunion	NDM-1	M	55
135I1	29/03/2017	Rectal swab	La Réunion	NDM-1	M	34
139C3	18/05/2017	deep sampling	Provence-Alpes-Côte d'Azur	CTX-M-15 ESBL + outermembrane decreased permeability	F	73
135B6	22/03/2017	Rectal swab	Provence-Alpes-Côte d'Azur	NDM-1	M	71
152A1	04/10/2017	Rectal swab	Ile-de-France	KPC-3	F	65
50D3	16/06/2014	Not determined	Ile-de-France	NDM-1	M	58
74D4	24/02/2015	Rectal swab	Ile-de-France	NDM-1	M	63
160J8	22/01/2018	Rectal swab	Ile-de-France	NDM-1	M	66
158G10	11/01/2019	Rectal swab	Auvergne-Rhône-Alpes	NDM-1	F	72
161D10	29/01/2018	Urine	Normandie	NDM-1	M	63
161E7	29/01/2018	Rectal swab	La Réunion	OXA-48	M	39
161H1	31/01/2018	Urine	Occitanie	NDM-1	M	71
150B5	25/09/2017	Urine	Occitanie	NDM-1	M	77
162C10	05/02/2018	Rectal swab	Occitanie	NDM-1	M	58
162D1	05/02/2018	Rectal swab	Occitanie	NDM-1	M	76
162D2	05/02/2018	Rectal swab	Occitanie	NDM-1	F	91
162D8	05/02/2018	Respiratory tract	Occitanie	NDM-1	M	91
151B5	04/10/2017	Rectal swab	Hauts-de-France	NDM-1	F	73
157A1	05/12/2017	Urine	Hauts-de-France	NDM-1	F	71
160E4	12/01/2018	Rectal swab	Ile-de-France	NDM-1 + OXA-48	F	59
162J9	12/02/2018	Rectal swab	Auvergne-Rhône-Alpes	OXA-181	M	70
162J10	12/02/2018	Urine	Ile-de-France	KPC-3	M	61
163E9	16/02/2018	Rectal swab	Hauts-de-France	NDM-5 + OXA-181	M	70
165G3	08/03/2018	Not determined	Hauts-de-France	NDM-1	M	70
166E4	15/03/2018	Rectal swab	Ile-de-France	OXA-181	M	49
166G6	19/03/2018	Rectal swab	Ile-de-France	NDM-1	F	62
166H3	20/03/2018	Rectal swab	Ile-de-France	NDM-1	F	61
167D3	27/03/2018	Urine	Occitanie	KPC-3	F	21
169G5	18/04/2018	Urine	Ile-de-France	NDM-1	M	52

170A3	20/04/2018	Rectal swab	Ile-de-France	NDM-5 + OXA-181	M	72
170A7	23/04/2018	Rectal swab	Ile-de-France	NDM-1	M	37
170D7	24/04/2018	Rectal swab	Provence-Alpes-Côte d'Azur	NDM-1	M	52
170D9	24/04/2018	Not determined	Provence-Alpes-Côte d'Azur	NDM-1	F	80
170G4	26/04/2018	Rectal swab	Ile-de-France	NDM-1	M	42
171E10	04/05/2018	Rectal swab	Ile-de-France	NDM-1 + OXA-48	M	78
174C5	04/06/2018	Urine	Auvergne-Rhône-Alpes	OXA-48	F	80
174D3	04/06/2018	Rectal swab	Ile-de-France	NDM-1 + OXA-48	F	68
176G4	29/06/2018	Rectal swab	Grand Est	NDM-1	M	18
177E9	06/07/2018	Rectal swab	Auvergne-Rhône-Alpes	KPC-3	F	78
181D10	07/08/2018	Rectal swab	Hauts-de-France	OXA-48	F	65
181H8	09/08/2018	Urine	Hauts-de-France	NDM-1	F	71
182F9	16/08/2018	Rectal swab	Hauts-de-France	NDM-1	M	69
182G1	16/08/2018	Urine	Hauts-de-France	NDM-1	F	91
182G4	16/08/2018	Rectal swab	Ile-de-France	NDM-1	M	44
183B1	21/08/2018	Urine	Centre — Val de Loire	OXA-48	F	118
183F8	23/08/2018	Rectal swab	Ile-de-France	KPC-3	M	65
183J4	27/08/2018	Rectal swab	Ile-de-France	NDM-1	M	62
183J8	27/08/2018	Rectal swab	Ile-de-France	NDM-1	M	68
184G7	31/08/2018	Rectal swab	Hauts-de-France	NDM-1	M	55
185G1	06/09/2018	Rectal swab	Centre — Val de Loire	KPC-3	M	67
186F4	12/09/2018	Other	Ile-de-France	NDM-1	M	43
186H6	14/09/2018	Rectal swab	Ile-de-France	KPC-3	F	80
187B6	18/09/2018	Rectal swab	Ile-de-France	NDM-5 + OXA-181	F	71
187D8	19/09/2018	Rectal swab	Hauts-de-France	NDM-1	F	87
188G4	27/09/2018	Rectal swab	Ile-de-France	NDM-1	M	62
188H6	28/09/2018	Urine	Nouvelle-Aquitaine	NDM-1	M	72
189D7	02/10/2018	Rectal swab	Grand Est	OXA-181	M	69
191B5	17/10/2018	Rectal swab	Auvergne-Rhône-Alpes	NDM-1	M	53
192F9	29/10/2018	Rectal swab	Bretagne	OXA-181	M	66
192I2	30/10/2018	Rectal swab	Ile-de-France	NDM-5 + OXA-181	M	90
194E7	13/11/2018	Rectal swab	Hauts-de-France	NDM-1	M	69
194H1	15/11/2018	Rectal swab	Hauts-de-France	NDM-1	F	56
195B8	20/11/2018	Rectal swab	Hauts-de-France	NDM-1	M	69
195C2	21/11/2018	Respiratory tract	Bourgogne-Franche-Comté	KPC-3	M	62
195G3	23/01/2018	Rectal swab	Ile-de-France	KPC-3	M	81
196G3	03/12/2018	Urine	Occitanie	NDM-1	M	71
196H9	04/12/2018	Rectal swab	Auvergne-Rhône-Alpes	NDM-1	M	24
196I3	04/12/2018	Urine	Auvergne-Rhône-Alpes	KPC-3	M	70
198B9	14/12/2018	Rectal swab	Centre — Val de Loire	KPC-3	M	68
198F6	18/12/2018	Rectal swab	Nouvelle-Aquitaine	OXA-48	F	64
198J6	21/12/2018	Rectal swab	Ile-de-France	NDM-1	M	53
199C6	26/12/2018	Urine	Ile-de-France	NDM-1	M	51
199D1	27/12/2018	Rectal swab	Centre — Val de Loire	NDM-4 + KPC-2	M	57
199D5	27/12/2018	Urine	Hauts-de-France	NDM-1	F	71
199D9	27/12/2018	Respiratory tract	Nouvelle-Aquitaine	OXA-48	F	82

199G5	31/12/2018	Rectal swab	Ile-de-France	NDM-1	F	64
200E3	08/01/2019	Urine	Hauts-de-France	NDM-1	F	82
200I6	10/01/2019	Rectal swab	Ile-de-France	NDM-1	M	79
201A1	11/01/2019	Rectal swab	Auvergne-Rhône-Alpes	NDM-1	F	71
201A5	11/01/2019	Rectal swab	Auvergne-Rhône-Alpes	NDM-1	F	62
201E4	15/01/2019	Urine	Occitanie	NDM-1	M	72
201E9	16/01/2019	Urine	Auvergne-Rhône-Alpes	NDM-1	F	91
201F10	16/01/2019	Rectal swab	Hauts-de-France	NDM-1	M	84
202B2	22/01/2019	Rectal swab	Provence-Alpes-Côte d’Azur	NDM-1	F	81
202B8	22/01/2019	Rectal swab	Hauts-de-France	NDM-1	M	89
202B9	22/01/2019	Rectal swab	Hauts-de-France	NDM-1	F	63
203D9	31/01/2019	Urine	Occitanie	NDM-1	M	72
203D10	31/01/2019	Rectal swab	Ile-de-France	NDM-5 + OXA-181	M	58
203H1	04/02/2019	Respiratory tract	La Réunion	NDM-1	F	67
204G2	13/02/2019	Rectal swab	Hauts-de-France	NDM-1	F	75
204G3	13/02/2019	Rectal swab	Ile-de-France	NDM-1	M	73
204I3	15/02/2019	Urine	Auvergne-Rhône-Alpes	NDM-1	F	76
205B10	20/02/2019	Rectal swab	Hauts-de-France	NDM-1	M	54
205E1	22/02/2019	Rectal swab	Hauts-de-France	NDM-1	M	64
205F9	26/02/2019	Other	Hauts-de-France	NDM-1	F	87
205F10	26/02/2019	Other	Hauts-de-France	NDM-1	M	58
206C3	01/03/2019	Rectal swab	Hauts-de-France	NDM-1	M	56
206I3	05/03/2019	Urine	Ile-de-France	NDM-1 + OXA-48	M	67
208A6	15/03/2019	Rectal swab	Hauts-de-France	NDM-1	M	77
208A7	15/03/2019	Rectal swab	Hauts-de-France	NDM-1	F	74
208B10	15/03/2019	Rectal swab	Ile-de-France	NDM-1	M	73
208E10	19/03/2019	Rectal swab	Hauts-de-France	NDM-1	M	88
208H1	20/03/2019	Rectal swab	Hauts-de-France	NDM-1	F	85
208H5	20/03/2019	Urine	Hauts-de-France	NDM-1	F	73
208J6	25/03/2019	Urine	Ile-de-France	NDM-1	M	74
209J2	01/04/2019	Rectal swab	Ile-de-France	NDM-5	M	67
209J5	01/04/2019	Urine	Nouvelle-Aquitaine	NDM-1	F	87
210A8	02/04/2019	Other	Hauts-de-France	NDM-1 + OXA-48	M	59
210E2	03/04/2019	Respiratory tract	Pays de la Loire	NDM-1	M	49
211B6	10/04/2019	Rectal swab	Occitanie	NDM-1	M	90
211G5	15/04/2019	Respiratory tract	Nouvelle-Aquitaine	OXA-48	F	84
211I1	16/04/2019	Rectal swab	Nouvelle-Aquitaine	NDM-1	F	87
211J5	17/04/2019	deep sampling	Hauts-de-France	NDM-1	F	65
212C10	19/04/2019	Rectal swab	Hauts-de-France	NDM-1	F	79
212F5	24/04/2019	Rectal swab	Hauts-de-France	NDM-1	M	96
212I1	25/04/2019	Urine	Provence-Alpes-Côte d’Azur	OXA-48	M	79
213F9	03/05/2019	Rectal swab	Nouvelle-Aquitaine	NDM-5 + OXA-181	M	70
214A4	07/05/2019	Rectal swab	Ile-de-France	OXA-48	M	73
214A8	07/05/2019	Rectal swab	Ile-de-France	NDM-1 + OXA-48	M	74
214G9	14/05/2019	Rectal swab	Auvergne-Rhône-Alpes	NDM-1	F	70
214I8	15/05/2019	Rectal swab	La Réunion	OXA-181	F	58

217H4	11/06/2019	Urine	Auvergne-Rhône-Alpes	KPC-3	M	64
218E8	13/06/2019	Rectal swab	Nouvelle-Aquitaine	OXA-48	F	83
218E10	13/06/2019	Urine	Nouvelle-Aquitaine	OXA-48	M	85
218I9	18/06/2019	Rectal swab	Ile-de-France	OXA-181	M	51
219C10	20/06/2019	Rectal swab	Hauts-de-France	NDM-1 + OXA-48	M	87
220H1	03/07/2019	Rectal swab	Auvergne-Rhône-Alpes	OXA-48	M	82
221I9	11/07/2019	Rectal swab	La Réunion	OXA-181	M	79
222A10	12/07/2019	Rectal swab	Hauts-de-France	NDM-1	M	52
222D2	15/07/2019	Rectal swab	Provence-Alpes-Côte d'Azur	NDM-1	F	83
224D8	30/07/2019	Rectal swab	Hauts-de-France	NDM-1	M	82
224H7	01/08/2019	Urine	Ile-de-France	NDM-1	M	87
226C6	14/08/2019	Rectal swab	Ile-de-France	NDM-1	F	79
226G6	19/08/2019	Rectal swab	La Réunion	NDM-1	M	72
227A5	20/08/2019	Rectal swab	Hauts-de-France	NDM-1	F	82
230A7	10/09/2019	Blood culture	Nouvelle-Aquitaine	NDM-1	M	53
230A8	10/09/2019	Rectal swab	Nouvelle-Aquitaine	NDM-1	F	52
230A9	10/09/2019	Rectal swab	Nouvelle-Aquitaine	NDM-1	M	66
230B4	10/09/2019	Blood culture	Bretagne	NDM-1	M	82
230F8	16/09/2019	Rectal swab	Hauts-de-France	NDM-1	M	66
230G2	16/09/2019	Rectal swab	Ile-de-France	OXA-48	F	57
231A8	18/09/2019	Rectal swab	Provence-Alpes-Côte d'Azur	NDM-1	F	76
231A9	18/09/2019	Respiratory tract	Provence-Alpes-Côte d'Azur	NDM-1	M	75
231B9	18/09/2019	Rectal swab	Ile-de-France	NDM-1	M	66
231D7	20/09/2019	Rectal swab	Hauts-de-France	NDM-1	M	65
231D10	20/09/2019	Rectal swab	Ile-de-France	NDM-1	F	23
233A1	04/10/2019	Blood culture	Nouvelle-Aquitaine	NDM-1	F	84
233B7	07/10/2019	Rectal swab	Ile-de-France	NDM-5	M	73
233D5	08/10/2019	Blood culture	Nouvelle-Aquitaine	NDM-1	F	74
233I1	14/10/2019	Rectal swab	Guyane	NDM-1	M	67
234J6	22/10/2019	Urine	Grand Est	NDM-1	M	56
235C9	24/10/2019	Blood culture	La Réunion	NDM-1	M	59
235E3	25/10/2019	Rectal swab	Auvergne-Rhône-Alpes	NDM-1	M	72
235G5	28/10/2019	Urine	Bretagne	OXA-48	M	78
235I10	30/10/2019	Urine	Hauts-de-France	NDM-1	M	71
236D6	04/11/2019	Other	La Réunion	NDM-1	M	119
237E4	13/11/2019	Rectal swab	Ile-de-France	NDM-1	F	18
238F7	19/11/2019	Rectal swab	Auvergne-Rhône-Alpes	NDM-1 + OXA-48	M	69
238G2	20/11/2019	Urine	Ile-de-France	NDM-1	F	66
238J5	21/11/2019	Rectal swab	Hauts-de-France	NDM-1	F	54
239A10	22/11/2019	Not determined	Grand Est	OXA-48	M	86
239B9	22/11/2019	Urine	Grand Est	OXA-48	M	88
239D10	25/11/2019	Rectal swab	Ile-de-France	NDM-1	M	56
240E5	05/12/2019	Rectal swab	Hauts-de-France	NDM-1	F	20
240F8	06/12/2019	Rectal swab	Ile-de-France	KPC-3	M	69
240G1	06/12/2019	Rectal swab	Hauts-de-France	NDM-1	M	56
241A7	11/12/2019	Rectal swab	Hauts-de-France	NDM-1	M	35

241D6	13/12/2019	Urine	Grand Est	OXA-48	M	72
242G10	26/12/2019	deep sampling	Guyane	NDM-1	F	73
242J2	30/12/2019	Urine	Occitanie	OXA-48	F	60
243C3	02/01/2020	Urine	Auvergne-Rhône-Alpes	KPC-3	F	80
247B1	06/02/2020	Rectal swab	Grand Est	NDM-1	M	75
247C4	07/02/2020	Rectal swab	Centre — Val de Loire	OXA-48	M	77
247D3	10/02/2020	Rectal swab	Auvergne-Rhône-Alpes	NDM-1	F	53
247H2	12/02/2020	Rectal swab	Guyane	NDM-1	M	62
248A10	14/02/2020	Urine	Grand Est	NDM-1	M	74
249A1	24/02/2020	Urine	Occitanie	NDM-1	M	91
245A10	21/01/2020	Rectal swab	Nouvelle-Aquitaine	NDM-1	F	51
249C2	25/02/2020	Rectal swab	Grand Est	NDM-1	M	70
249C5	25/02/2020	Rectal swab	Auvergne-Rhône-Alpes	NDM-1	M	75
249D6	25/02/2020	Urine	La Réunion	NDM-1	F	46
249F2	26/02/2020	Rectal swab	Occitanie	NDM-1	M	91
249G6	27/02/2020	deep sampling	Auvergne-Rhône-Alpes	OXA-48	M	74
249G8	27/02/2020	Rectal swab	Pays de la Loire	NDM-5 + OXA-181	M	73
249H7	28/02/2020	Rectal swab	Auvergne-Rhône-Alpes	NDM-1	M	66
250C4	04/03/2020	deep sampling	Auvergne-Rhône-Alpes	OXA-48	F	18
252C9	31/03/2020	Urine	Centre — Val de Loire	NDM-1	M	87
252D8	07/04/2020	Rectal swab	Hauts-de-France	CTX-M-3 ESBL + outermembrane decreased permeability	M	55
252F8	07/04/2020	Urine	Hauts-de-France	NDM-1	M	59
253A10	16/04/2020	Blood culture	Guyane	NDM-1	F	29
253E6	21/04/2020	Rectal swab	Nouvelle-Aquitaine	NDM-1	M	93
253F4	28/04/2020	Urine	Occitanie	NDM-1	M	93
253J10	06/05/2020	Rectal swab	Centre — Val de Loire	NDM-1	M	54
253G1	28/04/2020	Rectal swab	Hauts-de-France	CTX-M-15 ESBL + outermembrane decreased permeability	M	64
254B9	12/05/2020	Rectal swab	Ile-de-France	NDM-1	M	26
254C1	12/05/2020	Rectal swab	Ile-de-France	NDM-1	M	71
254G8	14/05/2020	Rectal swab	Ile-de-France	OXA-181	M	71
255B5	18/05/2020	Urine	Hauts-de-France	NDM-1	F	85
256B3	02/06/2020	Rectal swab	La Réunion	NDM-1	M	67
256D9	03/06/2020	Rectal swab	Ile-de-France	KPC-3	M	64
256G4	04/06/2020	Rectal swab	Bretagne	NDM-1	M	81
257C9	11/06/2020	Urine	Ile-de-France	KPC-3	M	84
257D7	12/06/2020	Urine	Centre — Val de Loire	NDM-1	M	68
O81I1	15/04/2020	00/01/1900	Not determined	CTX-M-15 ESBL + outermembrane decreased permeability	F	70
259D5	03/07/2020	Rectal swab	Ile-de-France	KPC-3	F	95
259D7	03/07/2020	Rectal swab	Ile-de-France	KPC-3	M	92
259D8	03/07/2020	Rectal swab	Ile-de-France	KPC-3	M	63
259D9	03/07/2020	Rectal swab	Ile-de-France	KPC-3	M	88
259G6	07/07/2020	Urine	Hauts-de-France	NDM-1	M	87
260E5	17/07/2020	Rectal swab	Auvergne-Rhône-Alpes	NDM-1	M	73
260F9	17/07/2020	Urine	Hauts-de-France	NDM-1	F	74
260G8	20/07/2020	Respiratory tract	Ile-de-France	OXA-181	M	59
261C2	24/07/2020	Rectal swab	Auvergne-Rhône-Alpes	NDM-1	M	87

261C7	27/07/2020	Rectal swab	Bourgogne-Franche-Comté	KPC-3	M	75
261H7	31/07/2020	Not determined	Bretagne	OXA-48	F	60
261J3	04/08/2020	Rectal swab	Hauts-de-France	NDM-1	F	83
262A5	05/08/2020	Rectal swab	Hauts-de-France	CTX-M-3 + CTX-M-15 ESBLs + outermembrane decreased permeability	M	59
262C7	06/08/2020	Rectal swab	Ile-de-France	KPC-3	M	76
262I6	12/08/2020	Blood culture	Provence-Alpes-Côte d'Azur	NDM-1	M	62
263E2	19/08/2020	Rectal swab	Ile-de-France	OXA-48	M	47
264A2	26/08/2020	Not determined	Occitanie	KPC-3	F	43
264D9	01/09/2020	Urine	Nouvelle-Aquitaine	NDM-1	M	80
265A2	09/09/2020	Rectal swab	Provence-Alpes-Côte d'Azur	NDM-1	M	87
265A10	09/09/2020	Rectal swab	Ile-de-France	KPC-3	M	83
265C5	11/09/2020	Rectal swab	Nouvelle-Aquitaine	NDM-1	M	72
265F10	15/09/2020	Urine	Hauts-de-France	NDM-1	F	81
265I1	17/09/2020	Rectal swab	Hauts-de-France	NDM-1	M	69
266A4	21/09/2020	Rectal swab	Ile-de-France	KPC-3	M	75
266D3	23/09/2020	Rectal swab	Ile-de-France	NDM-1	M	27
269B5	16/10/2020	Urine	Hauts-de-France	NDM-1	F	80
269I10	21/10/2020	Not determined	Auvergne-Rhône-Alpes	OXA-48	M	69
269J3	21/10/2020	Rectal swab	Centre — Val de Loire	NDM-1	M	65
269J4	21/10/2020	Rectal swab	Centre — Val de Loire	NDM-1	M	60
270C2	23/10/2020	Urine	Hauts-de-France	OXA-48	M	57
270E6	28/10/2020	Rectal swab	Centre — Val de Loire	NDM-1	M	59
270E7	28/10/2020	Rectal swab	Centre — Val de Loire	NDM-1	M	69
270E8	28/10/2020	Rectal swab	Centre — Val de Loire	NDM-1	F	68
270E9	28/10/2020	Rectal swab	Centre — Val de Loire	NDM-1	M	60
270I3	30/10/2020	Rectal swab	Ile-de-France	NDM-1	M	47
270I4	30/10/2020	Blood culture	Ile-de-France	NDM-1	M	64
270I5	30/10/2020	Rectal swab	Ile-de-France	NDM-5	M	51
271C4	04/11/2020	Urine	Occitanie	NDM-1	M	78
271C8	04/11/2020	Not determined	Hauts-de-France	CTX-M-15 ESBL + outermembrane decreased permeability	M	68
271I2	12/11/2020	Rectal swab	Auvergne-Rhône-Alpes	NDM-1	M	58
272D8	20/11/2020	Rectal swab	Centre — Val de Loire	NDM-1	M	55
272E10	23/11/2020	Urine	Nouvelle-Aquitaine	OXA-48	M	72
272H5	25/11/2020	Urine	Hauts-de-France	CTX-M-15 ESBL + outermembrane decreased permeability	M	56
273C4	01/12/2020	Urine	Occitanie	NDM-1	F	16
274D2	09/12/2020	Other	Provence-Alpes-Côte d'Azur	NDM-1	M	72
275C5	23/12/2020	Rectal swab	Ile-de-France	NDM-1	M	40
275I2	30/12/2020	Rectal swab	Ile-de-France	NDM-1	M	64
276G2	12/01/2021	Not determined	Centre — Val de Loire	NDM-1	F	67
277I1	25/01/2021	Rectal swab	Ile-de-France	OXA-181	M	63
278A10	27/01/2021	Urine	Grand Est	NDM-1	M	83
278C1	27/01/2021	Not determined	Guyane	CTX-M-15 ESBL + outermembrane decreased permeability	F	74
**279A3**	**09/02/2021**	**Rectal swab**	**Ile-de-France**	**NDM-14**	**M**	**83**
282A7	18/03/2021	Rectal swab	Grand Est	NDM-1 + OXA-48	F	75
282B6	19/03/2021	Rectal swab	Ile-de-France	OXA-181	F	37
282E1	23/03/2021	Rectal swab	Guyane	NDM-1 + OXA-48	M	82

285D2	03/05/2021	Rectal swab	Not determined	NDM-1 + OXA-48	M	57
285D10	04/05/2021	Urine	Ile-de-France	NDM-1	M	64
285E7	04/05/2021	Rectal swab	Ile-de-France	NDM-1	M	60
285E8	04/05/2021	Rectal swab	Ile-de-France	NDM-1	F	40
286C6	14/05/2021	Rectal swab	Ile-de-France	NDM-1	F	81
286F3	19/05/2021	Rectal swab	Guyane	OXA-48	M	78
286F7	19/05/2021	Blood culture	Nouvelle-Aquitaine	OXA-48	M	79
286G9	19/05/2021	Not determined	Pays de la Loire	NDM-1	M	41
286G10	19/05/2021	Not determined	Pays de la Loire	NDM-1	M	67
286H3	20/05/2021	Rectal swab	Ile-de-France	NDM-1	M	75
286H5	21/05/2021	Rectal swab	Auvergne-Rhône-Alpes	OXA-48	M	121
286I6	25/05/2021	Rectal swab	Ile-de-France	NDM-1	F	83
286J2	25/05/2021	Rectal swab	Ile-de-France	NDM-5 + OXA-181	M	56
287A6	26/05/2021	Rectal swab	Nouvelle-Aquitaine	OXA-48	F	78
287C10	28/05/2021	Rectal swab	Ile-de-France	NDM-1	M	88
287F2	31/05/2021	Rectal swab	Ile-de-France	NDM-5	M	37
287H2	02/06/2021	Urine	Ile-de-France	NDM-1	M	64
288D4	08/06/2021	Rectal swab	Guyane	OXA-48	M	91
288F10	10/06/2021	Rectal swab	Hauts-de-France	OXA-48	M	56
O89A1	15/04/2020	00/01/1900	Not determined	CTX-M-15 ESBL + outermembrane decreased permeability	F	67
288J2	15/06/2021	Urine	Hauts-de-France	OXA-48	M	89
289E9	22/06/2021	Rectal swab	Ile-de-France	NDM-1	M	121
290J8	06/07/2021	Urine	Provence-Alpes-Côte d'Azur	NDM-1	M	87
290J10	06/07/2021	Rectal swab	Provence-Alpes-Côte d'Azur	NDM-1	M	84
291A7	07/07/2021	Rectal swab	Ile-de-France	NDM-1	M	89
291B8	08/07/2021	Respiratory tract	Ile-de-France	NDM-1	F	62
291C6	08/07/2021	Rectal swab	Ile-de-France	NDM-1	M	66
291G9	13/07/2021	Rectal swab	Ile-de-France	OXA-181	M	60
291H1	13/07/2021	Urine	Occitanie	NDM-1	M	73
292J3	27/07/2021	Rectal swab	Guyane	OXA-48	F	58
292J4	27/07/2021	Urine	Nouvelle-Aquitaine	OXA-48	M	88
294B5	05/08/2021	Rectal swab	Ile-de-France	NDM-1	M	63
294F2	09/08/2021	Urine	Nouvelle-Aquitaine	NDM-1	F	89
294I10	13/08/2021	Urine	Nouvelle-Aquitaine	NDM-1	M	77
295A8	16/08/2021	Rectal swab	Ile-de-France	NDM-1	F	89
295B4	16/08/2021	Urine	Hauts-de-France	OXA-48	M	95
295B6	17/08/2021	Urine	Provence-Alpes-Côte d'Azur	NDM-1	M	86
295C2	17/08/2021	Rectal swab	Hauts-de-France	NDM-1	M	92
295G8	19/08/2021	Blood culture	Hauts-de-France	NDM-5	M	35
**296B5**	**25/08/2021**	**Rectal swab**	**Ile-de-France**	**NDM-14**	**M**	**41**
296B8	25/08/2021	Urine	Nouvelle-Aquitaine	OXA-48	M	88
291C2	08/07/2021	Rectal swab	Ile-de-France	NDM-1	M	73
296C9	25/08/2021	Rectal swab	Hauts-de-France	NDM-1	F	96
297B1	01/09/2021	Rectal swab	Hauts-de-France	NDM-1	M	62
297D2	03/09/2021	Rectal swab	Auvergne-Rhône-Alpes	OXA-48	F	75
292A6	19/07/2021	Rectal swab	Hauts-de-France	NDM-1	M	66

297H8	08/09/2021	Urine	Nouvelle-Aquitaine	NDM-1	F	89
**298G9**	**13/09/2021**	**Rectal swab**	**Centre — Val de Loire**	**NDM-14**	**M**	**72**
298J5	15/09/2021	Rectal swab	Ile-de-France	NDM-5	F	22
**299A10**	**16/09/2021**	**Rectal swab**	**Grand Est**	**NDM-14**	**M**	**81**
299E3	21/09/2021	Urine	Provence-Alpes-Côte d'Azur	NDM-1	M	85
300B2	27/09/2021	Urine	Grand Est	NDM-1	M	59
300B5	27/09/2021	Rectal swab	Ile-de-France	NDM-1	F	83
300H1	01/10/2021	Rectal swab	La Réunion	NDM-1	F	51
300I4	04/10/2021	Rectal swab	Ile-de-France	NDM-5	M	54
291C3	08/07/2021	Rectal swab	Ile-de-France	OXA-181	F	121
301D10	07/10/2021	Rectal swab	Guyane	NDM-1 + OXA-48	M	57
301H5	11/10/2021	Urine	Ile-de-France	NDM-1	F	76
299J8	23/09/2021	Rectal swab	Auvergne-Rhône-Alpes	NDM-1	M	70
300A1	23/09/2021	Urine	Auvergne-Rhône-Alpes	NDM-1 + OXA-48	F	63
298B9	09/09/2021	Rectal swab	Ile-de-France	NDM-1	M	75
302C7	13/10/2021	Urine	Hauts-de-France	NDM-1	M	83
**302F8**	**15/10/2021**	**Blood culture**	**Ile-de-France**	**NDM-14**	**M**	**74**
302I4	18/10/2021	Rectal swab	Guyane	NDM-1	F	78
303F10	25/10/2021	Other	Auvergne-Rhône-Alpes	NDM-1	M	56
304C6	28/10/2021	Urine	Hauts-de-France	NDM-1	M	63
305C6	10/11/2021	Rectal swab	Ile-de-France	NDM-1	F	79
305E8	15/11/2021	Rectal swab	Pays de la Loire	NDM-1	M	86
306D7	22/11/2021	Urine	Hauts-de-France	NDM-1	M	77
307B6	29/11/2021	Rectal swab	Ile-de-France	NDM-5	F	121
307H1	03/12/2021	Urine	Centre — Val de Loire	NDM-1	M	89
307J9	07/12/2021	Not determined	Ile-de-France	NDM-1	M	33
307J10	08/12/2021	Not determined	Ile-de-France	NDM-1	M	50
308A1	08/12/2021	Not determined	Ile-de-France	NDM-1	M	63
308B7	08/12/2021	Other	Auvergne-Rhône-Alpes	NDM-6	F	80
308D3	10/12/2021	Rectal swab	Ile-de-France	NDM-5	M	41
308E4	13/08/2021	Rectal swab	Nouvelle-Aquitaine	KPC-3	M	52
308I2	17/12/2021	Urine	Provence-Alpes-Côte d'Azur	NDM-1	M	75
308I3	17/12/2021	Urine	Provence-Alpes-Côte d'Azur	NDM-1	M	87
308I6	17/12/2021	Rectal swab	Hauts-de-France	NDM-1	M	74
309A5	20/12/2021	Rectal swab	Ile-de-France	NDM-5	M	39
**307A8**	**29/11/2021**	**Rectal swab**	**Ile-de-France**	**NDM-14**	**F**	**65**
309B5	21/12/2021	Urine	Ile-de-France	NDM-1	M	38
309D10	22/12/2021	Rectal swab	Auvergne-Rhône-Alpes	KPC-3	F	65
309F4	23/12/2021	Urine	Auvergne-Rhône-Alpes	NDM-1	F	88
309H6	24/12/2021	Rectal swab	Nouvelle-Aquitaine	NDM-1 + OXA-48	F	75
309I10	28/12/2021	Rectal swab	Provence-Alpes-Côte d'Azur	NDM-1	F	72
309J10	29/12/2021	Urine	Hauts-de-France	NDM-5	M	77
310B1	30/12/2021	Urine	Centre — Val de Loire	KPC-3	F	85
310F8	06/01/2022	Rectal swab	Provence-Alpes-Côte d'Azur	NDM-1	M	54
**310H4**	**07/01/2022**	**Urine**	**Not determined**	**NDM-14**	**M**	**92**
311A5	11/01/2022	Urine	Grand Est	NDM-1	M	84

311A10	12/01/2022	Blood culture	Auvergne-Rhône-Alpes	NDM-1	M	75
311C4	13/01/2022	Not determined	Ile-de-France	KPC-3	F	83
311E10	17/01/2022	Respiratory tract	Hauts-de-France	NDM-1	M	71
**311I4**	**19/01/2022**	**Rectal swab**	**Hauts-de-France**	**NDM-14**	**M**	**72**
**311I9**	**19/01/2022**	**Rectal swab**	**Not determined**	**NDM-14**	**F**	**71**
312C10	24/01/2022	Rectal swab	Ile-de-France	NDM-1	M	67
312E7	25/01/2022	Urine	Nouvelle-Aquitaine	NDM-1	M	90
312J6	28/01/2022	Urine	Ile-de-France	NDM-1	M	82
312J7	28/01/2022	Urine	Hauts-de-France	NDM-1	M	77
312J9	28/01/2022	Rectal swab	Ile-de-France	NDM-1	M	55
**313D10**	**02/02/2022**	**Respiratory tract**	**Ile-de-France**	**NDM-14**	**M**	**43**
235G10	28/10/2019	Urine	Hauts-de-France	OXA-232	M	80
**313H5**	**08/02/2022**	**Urine**	**Provence-Alpes-Côte d'Azur**	**NDM-14**	**M**	**92**
**314A2**	**09/02/2022**	**Urine**	**Corse**	**NDM-14**	**M**	**70**
**314A3**	**09/02/2022**	**Urine**	**Corse**	**NDM-14**	**M**	**92**
**314A4**	**09/02/2022**	**deep sampling**	**Corse**	**NDM-14**	**M**	**71**
314A6	09/02/2022	Rectal swab	La Réunion	NDM-1	F	64
314B2	10/02/2022	Urine	Auvergne-Rhône-Alpes	OXA-181	M	91
**314C7**	**14/02/2022**	**Urine**	**Ile-de-France**	**NDM-14**	**M**	**95**
314D1	14/02/2022	Rectal swab	Ile-de-France	NDM-1	M	85
**314H2**	**16/02/2022**	**Rectal swab**	**Corse**	**NDM-14**	**F**	**61**
**314H4**	**16/02/2022**	**Rectal swab**	**Corse**	**NDM-14**	**M**	**82**
**314H5**	**16/02/2022**	**Rectal swab**	**Corse**	**NDM-14**	**M**	**87**
**314H7**	**16/02/2022**	**Urine**	**Hauts-de-France**	**NDM-14**	**M**	**78**
**314H9**	**16/02/2022**	**Rectal swab**	**Corse**	**NDM-14**	**M**	**64**
314I6	16/02/2022	Urine	Ile-de-France	NDM-1	F	65
**315A8**	**21/02/2022**	**Rectal swab**	**Corse**	**NDM-14**	**M**	**79**
315B5	22/02/2022	Blood culture	Ile-de-France	OXA-181	M	65
315B8	22/02/2022	Rectal swab	Hauts-de-France	NDM-1	M	19
315B9	22/02/2022	Rectal swab	Ile-de-France	NDM-1	F	78
315F8	24/02/2022	Rectal swab	Provence-Alpes-Côte d'Azur	NDM-1	M	59
**316A1**	**01/03/2022**	**Rectal swab**	**Corse**	**NDM-14**	**M**	**14**
**316A9**	**01/03/2022**	**Urine**	**Corse**	**NDM-14**	**M**	**92**
316D7	07/03/2022	Rectal swab	Ile-de-France	NDM-1	F	74
316D8	07/03/2022	Rectal swab	Ile-de-France	NDM-1	F	122
316E2	07/03/2022	Rectal swab	Ile-de-France	NDM-1	M	74
316F7	07/03/2022	Rectal swab	Ile-de-France	NDM-1	F	72
**314H6**	**16/02/2022**	**Rectal swab**	**Corse**	**NDM-14**	**M**	**91**
**314H8**	**16/02/2022**	**Rectal swab**	**Corse**	**NDM-14**	**M**	**78**
**315G1**	**24/02/2022**	**Urine**	**Corse**	**NDM-14**	**F**	**43**
**315G2**	**24/02/2022**	**Blood culture**	**Corse**	**NDM-14**	**F**	**74**
**316J4**	**09/03/2022**	**Respiratory tract**	**Corse**	**NDM-14**	**M**	**78**
**317A3**	**09/03/2022**	**Rectal swab**	**Hauts-de-France**	**NDM-14**	**F**	**28**
317F10	15/03/2022	Rectal swab	Provence-Alpes-Côte d'Azur	NDM-1	M	67
317J3	15/03/2022	Urine	Nouvelle-Aquitaine	OXA-48	F	84
**319G10**	**01/04/2022**	**Urine**	**Provence-Alpes-Côte d'Azur**	**NDM-14**	**M**	**70**

319I10	04/04/2022	Rectal swab	Ile-de-France	NDM-5	F	9
**320B1**	**05/04/2022**	**Superficial sampling**	**Corse**	**NDM-14**	**M**	**92**
**320D10**	**07/04/2022**	**Respiratory tract**	**Corse**	**NDM-14**	**M**	**52**
**320F2**	**08/04/2022**	**Urine**	**Corse**	**NDM-14**	**F**	**19**
**320G1**	**11/04/2022**	**Rectal swab**	**Corse**	**NDM-14**	**M**	**71**
320G9	11/04/2022	Rectal swab	Hauts-de-France	OXA-48	M	74
321A3	13/04/2022	Rectal swab	Hauts-de-France	OXA-48	M	69
**321B6**	**13/04/2022**	**Urine**	**Corse**	**NDM-14**	**M**	**87**
321D3	13/04/2022	Siphon	Grand Est	NDM-1	M	122
321G10	20/04/2022	Rectal swab	Auvergne-Rhône-Alpes	NDM-1	M	78
321I2	20/04/2022	Rectal swab	Hauts-de-France	NDM-1 + OXA-48	M	69
321J3	21/04/2022	Blood culture	La Réunion	NDM-5	M	71
322A5	22/04/2022	Urine	Centre — Val de Loire	NDM-1	M	78
**322A6**	**22/04/2022**	**Urine**	**Corse**	**NDM-14**	**M**	**78**
322D1	26/04/2022	Rectal swab	Auvergne-Rhône-Alpes	NDM-1	M	84
322G7	27/04/2022	deep sampling	Ile-de-France	NDM-1	M	56
324C1	12/05/2022	Blood culture	Grand Est	NDM-1 + OXA-48	F	56
324C3	12/05/2022	Blood culture	Grand Est	NDM-1	F	56
324F4	16/05/2022	Rectal swab	Centre — Val de Loire	NDM-1	M	84
325G3	25/05/2022	Rectal swab	Provence-Alpes-Côte d’Azur	NDM-1	M	85
325H9	25/05/2022	Urine	Martinique	CTX-M-15 ESBL + outermembrane decreased permeability	M	83
326D1	25/05/2022	Rectal swab	Guyane	NDM-1 + OXA-48	F	48
**326J8**	**26/05/2022**	**Urine**	**Ile-de-France**	**NDM-14**	**F**	**84**

Table S2: Genome assembly quality metrics of the 37 ST-147 NDM-14-producing *K. pneumoniae* used in the study.

Strain	N50	L50	GC Content	Count Scaffold	Count Base	Mean Lenght	Median Length	MAPQ Mean	Mean Depth	Perc Deth Uper Than 30
**279A3**	159202	12	57.77	96	5268298	54878.10	18860.0	59.72	100.84	87.77
**296B5**	117898	15	57.70	131	5338037	40748.37	16269.0	59.66	108.32	84.69
**298G9**	92635	16	57.62	139	5383297	38728.75	13412.0	59.67	131.32	87.52
**299A10**	105970	14	57.56	117	5436878	46469.04	18181.0	59.71	119.84	83.68
**302F8**	99703	18	57.69	144	5363898	37249.29	17275.0	59.74	99.82	87.43
**307A8**	158896	12	57.26	125	5615991	44927.92	8692.0	59.66	124.52	82.72
**310H4**	105970	16	57.21	170	5665039	33323.75	7372.0	59.64	179.0	86.7
**311I4**	159078	13	56.85	119	5775828	48536.36	14457.0	59.48	114.81	88.44
**311I9**	138610	13	57.17	132	5638936	42719.21	9993.5	59.58	111.54	86.1
**313D10**	100007	16	57.35	155	5569740	35933.80	13951.0	59.73	126.07	85.09
**313H5**	197134	9	57.49	107	5484668	51258.57	10408.0	59.63	107.42	84.33
**314A2**	177565	10	57.36	110	5527357	50248.7	9860.5	59.5	102.64	83.54
**314A3**	192559	10	57.65	98	5331157	54399.56	13513.0	59.6	90.23	84.1
**314A4**	192559	10	57.32	115	5566610	48405.30	11591.0	59.5	98.79	80.75
**314C7**	94421	17	57.79	129	5268698	40842.62	17636.0	59.6	80.1	84.08
**314H2**	183893	11	57.07	119	5686528	47785.94	10048.0	59.5	161.48	87.01
**314H4**	152565	12	57.38	119	5535810	46519.41	13355.0	59.56	100.08	85.69
**314H5**	131843	11	57.29	119	5563883	46755.31	13081.0	59.47	116.59	84.25
**314H7**	99684	16	57.52	135	5468651	40508.52	16127.0	59.58	82.71	84.12
**314H9**	178184	10	57.41	109	5507228	50525.02	12448.0	59.46	110.45	87.41
**314H6**	123178	15	57.04	166	5860338	35303.24	6210.0	59.38	138.89	87.6
**314H8**	153878	13	56.83	123	5754383	46783.60	12533.0	59.66	143.51	85.68
**315A8**	140311	12	57.38	126	5547536	44028.06	10644.0	59.61	121.19	81.25
**315G1**	145280	12	56.92	149	5782657	38809.77	7354.0	59.64	161.99	84.1
**315G2**	158896	12	57.26	125	5615991	44927.92	8692.0	59.66	124.52	82.72
**316A1**	168965	12	57.21	115	5627885	48938.13	9309.0	59.55	127.17	84.84
**316A9**	129388	13	57.47	130	5510667	42389.74	9957.5	59.57	101.86	84.76
**316J4**	159346	11	56.81	148	5802447	39205.72	7385.0	59.5	214.63	88.92
**317A3**	129557	11	57.32	123	5560776	45209.56	11320.0	59.47	152.63	85.87
**319G10**	101578	14	56.95	163	5745694	35249.65	7596.0	59.59	159.59	85.69
**320B1**	82915	21	57.14	192	5668912	29525.58	8872.5	59.55	207.5	88.05
**320D10**	84338	22	57.21	187	5639324	30156.81	10964.0	59.65	196.93	89.28
**320F2**	65873	25	57.71	157	5339918	34012.21	22592.0	59.75	124.32	92.34
**320G1**	104034	18	56.74	157	5831322	37142.17	13875.0	59.56	108.39	89.29
**321B6**	147648	13	56.87	154	5780210	37533.83	9938.5	59.51	155.97	88.52
**322A6**	51397	32	57.86	203	5295359	26085.51	14457.0	59.76	62.45	82.96
**326J8**	57891	27	57.20	195	5662875	29040.38	13448.0	59.65	110.03	87.02
